# Effect of Metformin and Simvastatin in Inhibiting Proadipogenic Transcription Factors

**DOI:** 10.3390/cimb43030144

**Published:** 2021-11-25

**Authors:** Jelena Jakab, Milorad Zjalić, Štefica Mikšić, Ivan Tušek, Vesna Ćosić, Nikola Volarić, Dario Nakić, Aleksandar Včev, Blaženka Miškić

**Affiliations:** 1 Faculty of Medicine, Josip Juraj Strossmayer University of Osijek, Josipa Huttlera, 4, 31000 Osijek, Croatia; milzjalic@gmail.com (M.Z.); nvolaric@fdmz.hr (N.V.); 2 Faculty of Dental Medicine and Health, Josip Juraj Strossmayer University of Osijek, Osijek, Crkvena 21, 31000 Osijek, Croatia; stefica.miksic@fdmz.hr (Š.M.); vesna1.cosic@gmail.com (V.Ć.); avcev@fdmz.hr (A.V.); miskicblazenka@gmail.com (B.M.); 3 Faculty of Medicine, University of Novi Sad, Hajduk Veljkova 3, 21137 Novi Sad, Serbia; ivan.tusek@mf.uns.ac.rs; 4 Polyclinic Ćosić, Petra Preradovića 4, 35000 Slavonski Brod, Croatia; 5 Zadar General Hospital, Ul. Bože Peričića 5, 23000 Zadar, Croatia; 9dario.nakic@gmail.com; 6 General Hospital “Dr. Josip Benčević” Slavonski Brod, Ul. Andrije Štampara 42, 35000 Slavonski Brod, Croatia

**Keywords:** adipocytes, adipogenesis, obesity, metformin, simvastatin

## Abstract

Obesity is a multifactorial chronic disease characterized by the excessive accumulation of fat in adipose tissue driven by hypertrophy and hyperplasia of adipocytes through adipogenesis. Adipogenesis plays a key role in the development of obesity and related metabolic disorders, which makes it potential target for the therapeutic approach to obesity. An increasing number of studies confirm the pleiotropic action of the combined treatment with metformin and statins, suggesting their anti-hypertensive, anti-inflammatory, and anti-adipogenic effect. The aim of this study was to analyze the effect of different doses of metformin (MET) and simvastatin (SIM) on the expression of key transcription factors of adipogenesis. Mouse 3T3-L1 preadipocytes were induced to differentiation in adipogenic medium with sustained MET and SIM treatment to assess the effect on adipogenesis. Nine days after initiating adipogenesis, the cells were prepared for further experiments, including Oil Red O staining, RT-PCR, Western blotting, and immunocytochemistry. Treating the cells with the combination of MET and SIM slightly reduced the intensity of Oil Red O staining compared with the control group, and down-regulated mRNA and protein expression of PPARγ, C/EBPα, and SREBP-1C. In conclusion, the inhibitory effect of MET and SIM on adipocyte differentiation, as indicated by decreased lipid accumulation, appears to be mediated through the down-regulation of adipogenic transcription factors, peroxisome proliferator-activated receptor γ (PPARγ), CCAAT/enhancer binding pro-tein α (C/EBPα), and sterol regulatory element-binding protein 1 (SREBP-1C).

## 1. Introduction

Obesity is a multifactorial chronic disease characterized by the excessive accumulation of fat in adipose tissue and chronic inflammation with no visible infection or known autoimmune process [1]. It is a risk factor for the development of metabolic syndrome, cardiovascular disease (CVD), type 2 diabetes mellitus (T2DM), hypertension, dyslipidemia, and malignancies [2]. Adipose tissue is the main regulator of energy balance and glucose homeostasis in a healthy body [3]. Excessive expansion of white adipose tissue (WAT) occurs due to an imbalance between caloric intake and energy expenditure leading to an increase in the size (hypertrophy) and number (hyperplasia) of adipocytes [4]. Adipocyte hypertrophy promotes the development of a chronic pro-inflammatory condition and infiltration of macrophages and leukocytes into adipose tissue, which further enhances the inflammatory condition. Adipocyte hyperplasia acts as a protective factor for metabolic overload of adipocytes [5]. Adipogenesis is a multistep process regulating the development of adipose cells, which includes proliferation of precursor cells, commitment to the adipogenic lineage, and terminal differentiation [6]. It is an important determinant of adipocyte count and total adipose tissue volume, with a key role in the development of obesity and related metabolic disorders [7]. Therefore, understanding the complex interaction of metabolic pathways during adipogenesis is important in finding potential targets for the therapeutic approach to obesity.

Treatment of obesity is still the subject of research because existing drugs do not have sufficient physiological specificity and cause a lot of side effects. The discovery of new drugs that could regulate adipocyte size, number, or function would greatly contribute to obesity prevention and control. Adipocyte cell cultures have become an indispensable part of research focused on adipocyte pathophysiology and energy metabolism. Differentiation of multipotent 3T3-L1 fibroblasts into mature adipocytes is one of the most commonly used in vitro models for studying adipose tissue biology. After initial treatment with insulin (INS), dexamethasone (DEX), and 1-methyl-3-isobutyl-xanthine (IBMX), increased adipogenic gene expression leads to increased triglyceride synthesis, and cells begin to accumulate lipid droplets [8]. Lately, clinically used pharmaceuticals for treating obesity-related diseases have shown body weight lowering effects, thus becoming a target for in vitro studies on adipogenesis.

Metformin (MET) is the most commonly used drug for the treatment of T2DM. The mechanism of action includes suppression of hepatic glucose production and improvement of adipose tissue metabolism in the liver and muscles, leading to a decrease in plasma glucose levels [9]. Metformin has a pleiotropic effect in reducing appetite, preventing CVD, improving endothelial function, modulating inflammation, and preventing cancer [2]. The anti-aging effect in humans has been revealed in experimental studies and reported in humans, with significantly lower all-cause mortality in diabetic subjects taking metformin than in non-diabetic subjects [10]. Metformin can suppress various pathological mechanisms implied in the aging process, such as inflammation inhibition, and balancing the amount of endogenous reactive oxygen species. It is suggested that metformin can exert anti-aging effects through AMP-activated protein kinase (AMPK) activation and mTOR Complex 1 (mTORC1) suppression [11]. The same mechanism is responsible for anti-cancer effects of metformin, as suggested in one study that evaluated the safety and activity of metformin combined with erlotinib in second-line treatment of patients with stage IV non-small cell lung cancer (NSCLC) [12]. MET therapeutic concentrations may improve vascular endothelial reactivity in non-diabetic patients, regardless of glucose levels [13]. Metformin may induce AMPK-dependent phosphorylation of endothelial nitric oxide synthase (NOS), thus resulting in increased nitric oxide (NO) production and vasodilation [14]. The effect of metformin on endocrine adipose tissue function is still debated, given that it has been shown to be more effective in obese diabetic patients than in those with lower body mass index (BMI) [15]. Although clinical studies show the effect of MET in weight loss, data on the effect of MET on adipogenesis in vitro are scarce.

Statins act as competitive inhibitors of 3-hydroxy-3-methylglutaryl coenzyme-A reductase, thereby reducing cholesterol production and intracellular cholesterol levels in hepatocytes. Statins, through their antioxidant and anti-inflammatory effects, reduce cardiovascular risk [16] and lipotoxicity by lowering total cholesterol and low-density lipoproteins (LDL), and by raising high-density lipoprotein (HDL) levels. The idea of using both MET and statins to assess the effect on the adipogenesis came based on the clinical studies. In one study conducted on 41 patients with a BMI > 25 kg/m^2^, MET 1.7 g/day for 16 weeks significantly reduced BMI and waist circumference, and simvastatin (SIM) 20 mg/day significantly reduced LDL and triglycerides levels [17]. An increasing number of studies confirm the pleiotropic action of the combined treatment with MET and statins, suggesting that such treatment might be useful in some diseases due to cardioprotective, anti-inflammatory, and anti-adipogenic effect [18,19].

The aim of this study was to analyze the effect of different doses of MET and SIM on the expression of key transcription factors of adipogenesis, peroxisome proliferator-activated receptor γ (PPARγ), CCAAT/enhancer binding protein α (C/EBPα), and sterol regulatory element-binding protein 1 (SREBP-1C).

## 2. Materials and Methods

### 2.1. Drugs and Reagents

Metformin (MET), simvastatin (SIM), isobutyl-3-methylxanthine (IBMX), dexamethasone (DEX), and insulin (INS) were purchased from Sigma-Aldrich (St. Louis, MO, USA). Dulbecco’s modified Eagle’s medium (DMEM) and fetal bovine serum (FBS) were purchased from Sigma-Aldrich (St. Louis, MO, USA), and Penicillin/Streptomycin (P/S) was obtained from Capricorn Scientific GmbH (Ebsdorfergrund, Hessen, Germany). Primary antibodies against SREBP-1C and STAT3 were obtained from Abcam (Cambridge, UK), a primary antibody against SMAD3 was obtained from NSJ Bioreagents (San Diego, CA, USA), against PPARγ from Sigma-Aldrich (St. Louis, MO, USA), and C/EBPα from Antibodies-online.com (Ebsdorfergrund, Hessen, Germany). HRP-conjugated GAPDH Monoclonal antibody was obtained from Proteintech (Rosemont, IL, USA). Secondary anti-rabbit antibody labeled with HRP and the biotinylated goat anti-rabbit IgG (H+L) secondary antibody were obtained from Jackson ImmunoResearch (West Grove, PA, USA). BD anti-Streptavidin, PE-Cy™5 was purchased from Fisher Scientific (Hampton, NH, USA).

### 2.2. Cell Culture and 3T3-L1 Cell Differentiation

Mouse 3T3-L1 preadipocytes (Elabscience Biotechnology Inc., Houston, TX, USA) were maintained in DMEM containing 4.5 g/L glucose supplemented with 10% FBS, 1% penicillin/streptomycin, and 15 mM HEPES (basal medium) at 37 °C in a humidified incubator with 5% CO_2_. Two days after reaching confluence, 3T3-L1 preadipocytes were induced to differentiation in adipogenic medium containing 10% FBS, 100 units penicillin and 0.01 mg streptomycin per mL, 15 mM HEPES, 10 μg/mL INS, 0.5 mM IBMX, and 1 μM DEX. Cells were incubated in adipogenic medium for three days and then cultured in medium containing 10% FBS, 100 units penicillin, and 0.01 mg streptomycin per mL, 15 mM HEPES, and 10 μg/mL INS for the following three days. Cells were then maintained in basal medium for additional three days. To assess the effects of MET and SIM on adipogenesis, the cells were treated during all nine days of the adipogenesis process with MET (200 µM, 2 mM, 4 mM), SIM (100 nm, 1 µM, 2 µM), and the MET+SIM combination therapy (SIM 100 nm + MET 200 µM, SIM 1 µM + MET 2 mM, SIM 2 µM + MET 4 mM). Ten days after initiating adipogenesis, the cells were prepared for further experiments, including Oil Red O staining, PCR, Western blotting, and immunocytochemistry.

### 2.3. Cell Viability Assay

3T3-L1 preadipocytes were seeded in a 96-well plate at a density of 1 × 10^5^ cells/mL and maintained following previously described protocol and conditions for cell differentiation. After incubation, 10 μL of 3-(4,5-dimethylthiazol-2yl)-2,5-diphenyl tetrazolium bromide (MTT) stock solution (5 mg mL/L) was added to each well, and the plates were incubated at 37° for 4 h. After the incubation, the formazan crystals were dissolved by 100 μL of MTT solvent prepared according to Sigma Aldrich instructions. Absorbance was read at 595 nm using an iMark microplate reader (Bio-rad, Hercules, CA, USA). The data are shown as a percentage of cell viability relative to the control group.

### 2.4. Oil Red O Lipid Staining

3T3- L1 cells were differentiated in 6-well plates with cover slides previously prepared following the protocol by Zjalić et al. [20] and coated with Poly-D-Lysine (Sigma Aldrich, St. Louis, MO, USA). On day 10 of differentiation, the medium was removed, and cells were fixed with 2% formalin for 30 min at +4 °C. After fixation, cells were washed and stored in 1× PBS until further use. Oil Red O was prepared as a 0.5% stock solution in isopropanol, and a working Oil Red O solution as 40% water and 60% Oil Red O stock solution. Fixed cells were stained with working solution for 30 min. After rinsing two times with Phosphate Buffered Saline (PBS), slides were mounted in the Fluorescent mounting medium with 4′,6-diamidino-2-phenylindole (DAPI) (Abcam, Cambridge, UK). The cells were visualized using Axioskop 2 MOT microscope with mounted Olympus D70 camera, controlled through computer program DP Manager 1.2.1.107 and DP Controller 1.2.1.108. ImageJ-Fiji software was used to count cell nuclei and measure integrated density relative to the cell count.

### 2.5. Gene Expression Analysis

The 3T3-L1 preadipocytes cells were differentiated in 6-well plates and harvested on day 10 of differentiation. Total RNA was extracted using TRIzol reagent (Sigma-Aldrich, St. Louis, MO, USA). After extraction, the RNA was quantified using a NanoPhotometer^®^ P-Class P330-30 microvolume spectrophotometer (Implen, Germany), reporting concentration (in ng/μL) and sample purity (as 260/280 absorbance). First-strand cDNA synthesis was completed using a High-Capacity Reverse Transcriptase kit (Applied Biosystems, USA) according to the manufacturer’s protocol. RT-PCR was performed using Taq PCR Core Kit (Qiagen, Hilden, Germany) on DNA Engine^®^ Thermal Cycler (Bio-Rad, Hercules, CA, USA). After 3 min polymerase activation at 94 °C, 30 cycles with 94 °C for 45 s (denaturation), annealing for 45 s at annealing temperature depending on primers, and 70 °C for 1 min (extension) were performed, followed by finishing step at 70 °C for 10 min. The primer sequences used for PCR were as follows: PPARγ 5′-GCATGGTGCCTTCGCTGA-3′ (forward) and 5′-TGGCATCTCTGTGTCAACCATG-3′ (reverse); C/EBPα 5′-CAAGAACAGCAACGAGTACCG-3′ (forward) and 5′-GTCACTGGTCAACTCCAGCAC-3′ (reverse); SREBP-1C 5′-GCGGTTGGCACAGAGCTT-3′ (forward) and 5′-CTGTGGCCTCATGTAGGAATACC-3′ (reverse); ACTB 5′-CCTGTGCTGCTCACCGAGGC-3′ (forward); and 5′-GACCCCGTCTCTCCGGAGTCCATC-3′ (reverse). All primers were provided by Metabion International AG (Planegg, Germany). Agarose gel electrophoresis was used to separate DNA fragments which were visualized using the ChemiDoc™ Imaging system (Bio-Rad, Hercules, CA, USA) after staining with Diamond™ Nucleic Acid Dye (Promega, Madison, WI, USA). ImageJ-Fiji software was used to quantitatively analyze signals of the investigated genes. Gene expression was normalized to the expression of the housekeeping gene Actin Beta (ACTB) and further analyzed.

### 2.6. Protein Extraction and Western Blot

After differentiation in 6-well plates, cells were scraped, transferred in a 1.5 mL Eppendorf tube and pelleted in a centrifuge at 130× *g* for 5 min at 4 °C. Homogenization buffer consisted of 1× PBS, 0.32M sucrose, 5 mM sodium fluoride (NaF), 1mM each of ethylenediaminetetraacetic acid (EDTA), phenylmethylsulfonyl fluoride (PMSF), sodium orthovanadate (Na_3_VO_4_) (Sigma-Aldrich, St. Louis, MO, USA), and complete mini protease inhibitor (1 tablet per 10 mL of buffer) (Roche, Basel Switzerland), was added to the pellet. Cells were homogenized on ice with an ultrasonic homogenizer Bandelin Sonopuls 2070 (BANDELIN electronic GmbH & Co. KG, Berlin, Germany), and the homogenate was centrifuged for 15 min at 1000× *g* at 4 °C. Pellet was discarded, and supernatant was used in further analysis. Supernatant protein content was measured using Bradford protein assay on the iMark microplate reader at 595 nm. Sample aliquots were diluted to 0.5 mg/mL with 1× PBS buffer and mixed with western blot sample buffer in a 1:5 ratio. Samples were heated up to 100 °C for 5-min and stored at 4 °C. Prepared proteins were separated by sodium dodecyl sulfate (SDS)-polyacrylamide gel (12%) electrophoresis in Hoeffer mighty small electrophoresis system (Hoeffer Inc., San Francisco, CA, USA) with a continuous current of 15 mA per gel. Separated proteins were transferred to polyvinylidene difluoride (PVDF) membranes in TE22 Mighty small transfer tank (Thermo Fisher Scientific, Waltham, A, USA). Nonspecific reactions were blocked by a solution of 3% bovine serum albumin (BSA) in 1× PBS buffer with 0.1% Tween 20 detergent (PBST). Membranes were incubated with the primary antibody solution overnight at +4 °C. Membranes were washed 3 times for 10 min in PBST buffer and then incubated with horseradish peroxidase (HRP)-conjugated secondary antibody (Jackson ImmunoResearch, 1:20,000) for 2 h at room temperature. Specific proteins were detected using a chemiluminescent detection solution Immobilon^®^ Forte Western HRP Substrate (Millipore, Burlington, MA, USA) using the ChemiDoc™ Imaging system. Glyceraldehyde 3-phosphate dehydrogenase (GAPDH) was used as an internal control. ImageJ-Fiji software was used to quantitatively analyze signals of the investigated proteins.

### 2.7. Immunocytochemistry and Immunofluorescent Staining

3T3- L1 cells were differentiated in 6-well plates with cover slides previously prepared following the protocol by Zjalić et al. [20] and coated with Poly-D-Lysine (Sigma Aldrich, St. Louis, MO, USA). On day 10 of differentiation, the medium was removed and cells were fixed with 2% formalin for 30 min at 4 °C. After rinsing with 1× PBS, cover slides were transferred to blocking solution. After incubation for 4 h, a blocking solution was removed and primary antibody solution was added. After 48 h, cover slides were washed three times for 10 min with 1× PBS. The secondary antibody solution was added and incubated for 4 h at 4 °C. Cover slides were washed three times for 10 min with 1× PBS, followed by 1 h incubation in streptavidin labeled with a fluorophore. Cover slides were again washed three times for 10 min in precooled 1× PBS. Slides were mounted in the Fluorescent mounting medium with DAPI and captured on the Axioskop 2 MOT microscope. Protein expression levels were quantified using ImageJ-Fiji software, as immunostaining intensity relative to the cell count. The ratio of average pixel intensity in the nucleus over average pixel intensity of the cytosol was termed nuclear: cytoplasmic ratio and compared between experimental conditions and the control group.

### 2.8. Statistical Analysis

Data were analyzed in Past 4.06b, free software for scientific data analysis [21], using one-way ANOVA with Post-hoc Tukey HSD. Results were presented as mean ± SD; a probability level of * *p*-value < 0.05 was regarded as statistically significant.

## 3. Results

### 3.1. Effects of Metformin and Simvastatin on Cell Viability and Lipid Accumulation

**Figure 1:**
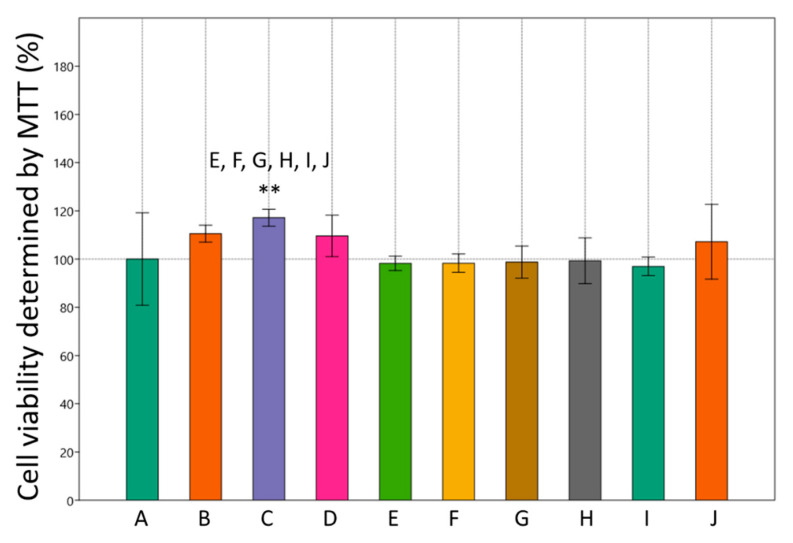
Effects of metformin and simvastatin on cell viability of 3T3-L1 cells, determined by MTT. A = differentiated cells, B = cells treated with metformin 200 µM, C = cells treated with metformin 2 mM, D = C = cells treated with metformin 4 mM, E = cells treated with simvastatin 100 nM, F = cells treated with simvastatin 1 µM, G = cells treated with simvastatin 2 µM, H = cells treated with the combination of metformin 200 µM and simvastatin 100 nM, I = cells treated with the combination of metformin 2 mM and simvastatin 1 µM, J = cells treated with the combination of metformin 4 mM and simvastatin 2 µM. The data are shown as the means ± SD (standard deviation) from three independent experiments. Data represents a percentage relative to differentiated cells as the control group (One-way ANOVA _F(9,29) = 6.355, *p* = 0.0002922_ with Tukey HSD post hoc test; ** *p* < 0.01). Asterisk (** *p* < 0.01) above the bars represent statistically significant differences of tested group in comparison to the control group. To highlight the differences between tested groups, every tested group was labeled with letter (A)–(J). Above the bars, there are letters of other tested groups where significant differences were found relative to the tested group belonging to a certain bar (x = *p* < 0.05, X = *p* < 0.01, X = *p* < 0.001).

To assess the effect of MET and SIM on adipogenesis, we first analyzed their effect on cell viability. MTT assay results showed that neither metformin nor simvastatin produced a toxic effect in all three concentrations, even when used as a combined treatment (Figure 1).

**Figure 2:**
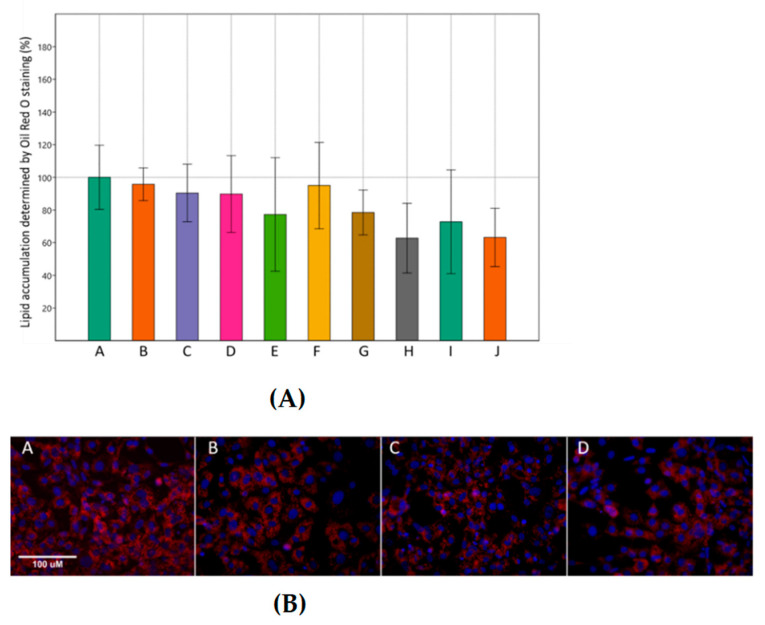
Effects of metformin and simvastatin on lipid accumulation in 3T3-L1 cells. (**A**) The integrated density of quantified Oil Red O staining relative to the cell count. A = differentiated cells, B = cells treated with metformin 200 µM, C = cells treated with metformin 2 mM, D = C = cells treated with metformin 4 mM, E = cells treated with simvastatin 100 nM, F = cells treated with simvastatin 1 µM, G = cells treated with simvastatin 2 µM, H = cells treated with the combination of metformin 200 µM and simvastatin 100 nM, I = cells treated with the combination of metformin 2 mM and simvastatin 1 µM, J = cells treated with the combination of metformin 4 mM and simvastatin 2 µM. The data are shown as the means ± SD (standard deviation) from three independent experiments. Data represents a percentage relative to differentiated cells as the control group (One-way ANOVA _F(9,27) = 1.04, *p* = 0.4474_ with Tukey HSD post hoc test); (**B**) representative microscopic images presenting lipid droplets formation visualized by Oil Red O staining, captured on the Axioskop 2 MOT microscope, 400×, scale 100 µM. Blue = DAPI, cell nuclei; red = Oil Red O, lipid droplets. A = differentiated cells, B = cells treated with the combination of metformin 200 µM and simvastatin 100 nM, C = cells treated with the combination of metformin 2 mM and simvastatin 1 µM, D = cells treated with the combination of metformin 4 mM and simvastatin 2 µM.

Incubation of 3T3-L1 cells in adipogenic medium induced cell differentiation with an increase in lipid droplet formation. Combined treatment with MET and SIM reduced the lipid droplet formation, although this reduction was not statistically significant (Figure 2A). On day 10, as shown in Figure 2B, treating the cells with the combination of MET and SIM reduced the intensity of Oil Red O staining compared with the control group. 

### 3.2. Effects of Metformin and Simvastatin on Expression of Adipogenic Genes

**Figure 3:**
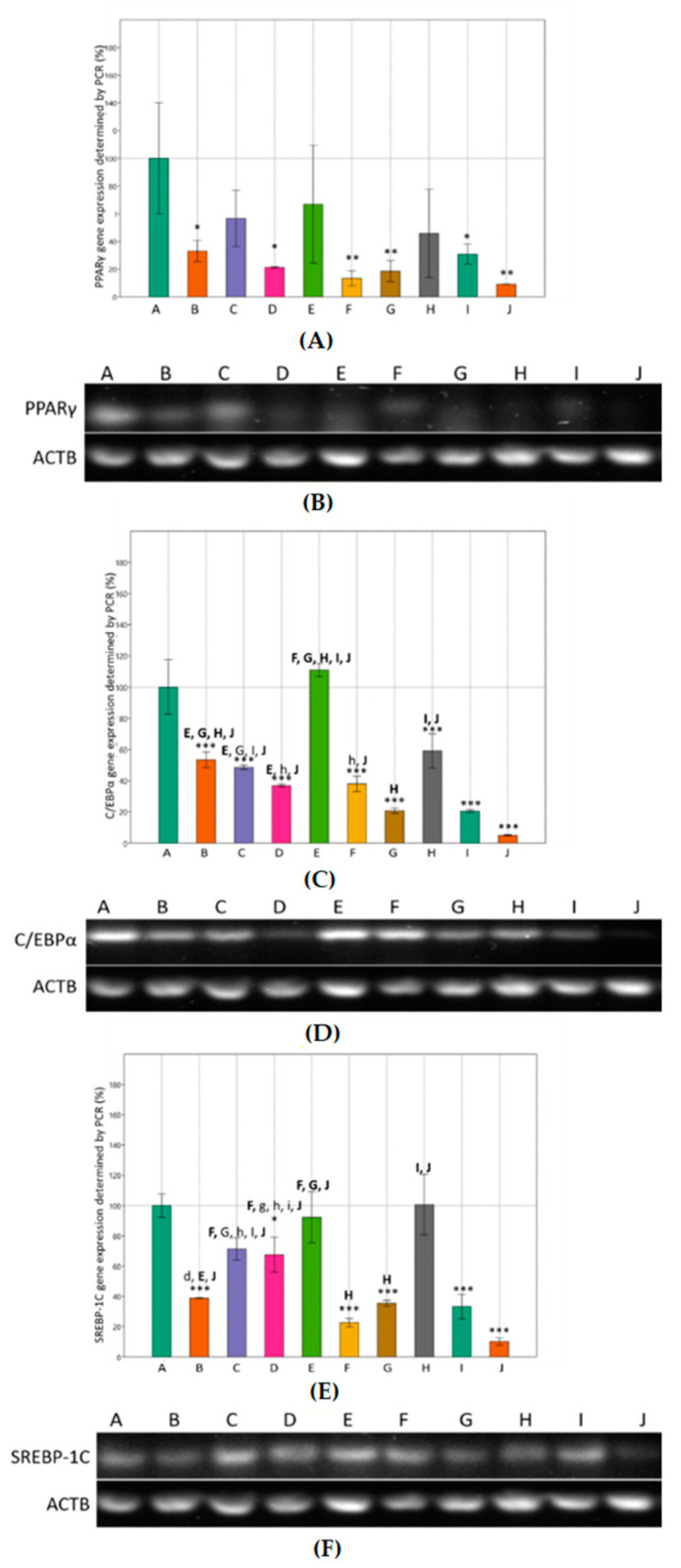
Effects of metformin and simvastatin on expression of adipogenic genes in 3T3-L1 cells, determined by RT-PCR. A = differentiated cells, B = cells treated with metformin 200 µM, C = cells treated with metformin 2 mM, D = C = cells treated with metformin 4 mM, E = cells treated with simvastatin 100 nM, F = cells treated with simvastatin 1 µM, G = cells treated with simvastatin 2 µM, H = cells treated with the combination of metformin 200 µM and simvastatin 100 nM, I = cells treated with the combination of metformin 2 mM and simvastatin 1 µM, J = cells treated with the combination of metformin 4 mM and simvastatin 2 µM, PPARγ = peroxisome proliferator-activated receptor γ, C/EBPα = CCAAT/enhancer binding protein α, SREBP-1C = sterol regulatory element-binding protein 1, ACTB = Actin Beta. Gene expression was normalized to the expression of the housekeeping gene ACTB. The data are shown as the means ± SD (standard deviation) from three independent experiments. Data represents a percentage relative to differentiated cells as the control group. Asterisk (* *p* < 0.05, ** *p* < 0.01, *** *p* < 0.001) above the bars represent statistically significant differences of tested group in comparison to the control group. To highlight the differences between tested groups, every tested group was labeled with letter (A)–(J). Above the bars there are letters of other tested groups where significant differences were found relative to the tested group belonging to a certain bar (x = *p* < 0.05, X = *p* < 0.01, X = *p* < 0.001). (**A**,**B**) Effects of metformin and simvastatin on expression of PPARγ during the differentiation of 3T3-L1 cells (One-way ANOVA _F(9,29) = 4802, *p* = 0.001687_ with Tukey HSD post hoc test); (**C**,**D**) effects of metformin and simvastatin on expression of C/EBPα during the differentiation of 3T3-L1 cells (One-way ANOVA _F(9,29) = 69.12, *p* = 4.26 × 10^−13^_ with Tukey HSD post hoc test;); (**E**,**F**) Effects of metformin and simvastatin on expression of SREBP-1C during the differentiation of 3T3-L1 cells (One-way ANOVA _F(9,29) = 32.91, *p* = 4.56 × 10^−10^_ with Tukey HSD post hoc test).

We examined whether MET and SIM affected the mRNA levels of adipogenic transcription factors PPARγ, C/EBPα, and SREBP-1C during adipocyte differentiation. Figure 3 shows the effect of sustained MET and SIM exposure for nine days on the gene expression levels. Compared to differentiated cells, significant down-regulation of C/EBPα and SREBP-1C was observed upon treatment with the highest concentration of combined MET-SIM treatment (4 mM + 2 µM), but also when treating cells only with SIM in higher concentration (1 µM and 2 µM) (Figure 3B,C). The same effect was observed on PPARγ mRNA levels, only not statistically significant. On the other hand, the lowest concentration of SIM (100 nM) and combined MET-SIM treatment (100 nM + 200 µM) had similar effects on all examined transcription factors, where the mRNA levels were equal or even slightly increased compared to differentiated cells (Figure 3).

### 3.3. Effects of Metformin and Simvastatin on Protein Expression and Immunoreactivity of Adipogenic Transcription Factors

**Figure 4:**
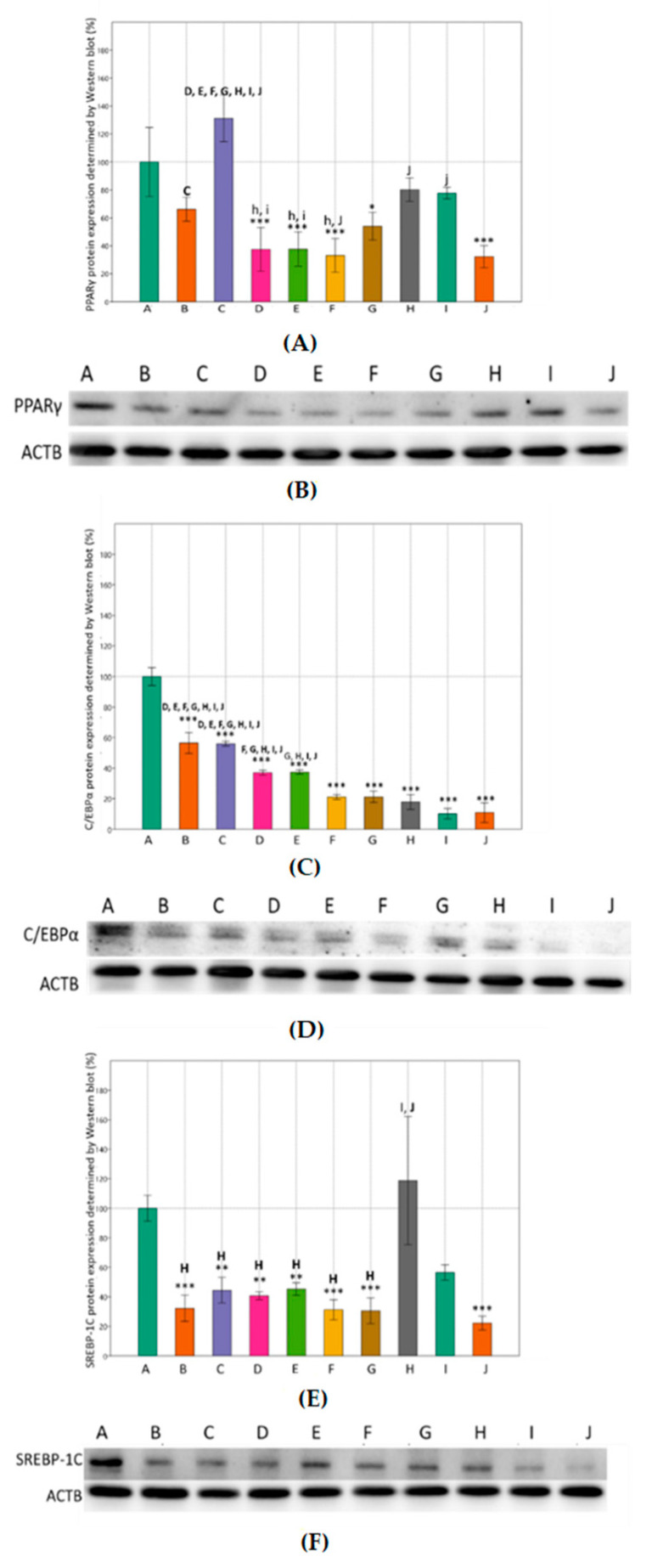
Effects of metformin and simvastatin on adipogenic protein expression in 3T3-L1 cells, determined by Western blot. A = differentiated cells, B = cells treated with metformin 200 µM, C = cells treated with metformin 2 mM, D = C = cells treated with metformin 4 mM, E = cells treated with simvastatin 100 nM, F = cells treated with simvastatin 1 µM, G = cells treated with simvastatin 2 µM, H = cells treated with the combination of metformin 200 µM and simvastatin 100 nM, I = cells treated with the combination of metformin 2 mM and simvastatin 1 µM, J = cells treated with the combination of metformin 4 mM and simvastatin 2 µM, PPARγ = peroxisome proliferator-activated receptor γ, C/EBPα = CCAAT/enhancer binding protein α, SREBP-1C = sterol regulatory element-binding protein 1, GAPDH = Glyceraldehyde 3-phosphate dehydrogenase. Results were normalized to GAPDH expression levels. The data are shown as the means ± SD (standard deviation) from three independent experiments. Data represents a percentage relative to differentiated cells as the control group. Asterisk (* *p* < 0.05, ** *p* < 0.01, *** *p* < 0.001) above the bars represent statistically significant differences of tested group in comparison to the control group. To highlight the differences between tested groups, every tested group was labeled with letter (A)–(J). Above the bars there are letters of other tested groups where significant differences were found relative to the tested group belonging to a certain bar (x = *p* < 0.05, X = *p* < 0.01, X = *p* < 0.001). (**A**,**B**) Effects of metformin and simvastatin on PPARγ protein expression during the differentiation of 3T3-L1 cells (One-way ANOVA _F(9,29) = 18.52, *p* = 7.61 × 10^−8^_ with Tukey HSD post hoc test); (**C**,**D**) effects of metformin and simvastatin on C/EBPα protein expression during the differentiation of 3T3-L1 cells (One-way ANOVA _F(9,29) = 129.1, *p* = 9.97 × 10^−16^_ with Tukey HSD post hoc test); (**E**,**F**) effects of metformin and simvastatin on SREBP-1C protein expression during the differentiation of 3T3-L1 cells (One-way ANOVA _F(9,29) = 13.26, *p* = 1.24 × 10^−6^_ with Tukey HSD post hoc test).

The protein expression levels of key regulators in adipogenesis were further analyzed. The effects of MET and SIM on the protein expression of the adipogenic transcription factors was examined using Western blot analysis and immunocytochemistry. Consistent with our results on gene expression, the highest concentration of combined MET-SIM treatment markedly decreased expression of examined proteins compared to differentiated cells (Figure 4). The exposure of cells to MET and SIM had the most effect on C/EBPα protein expression which decreased in a dose-dependent manner compared to the control group. Overall, the protein expression of all adipogenic markers determined by Western blot was decreased in almost every group of treated cells compared to control.

**Figure 5:**
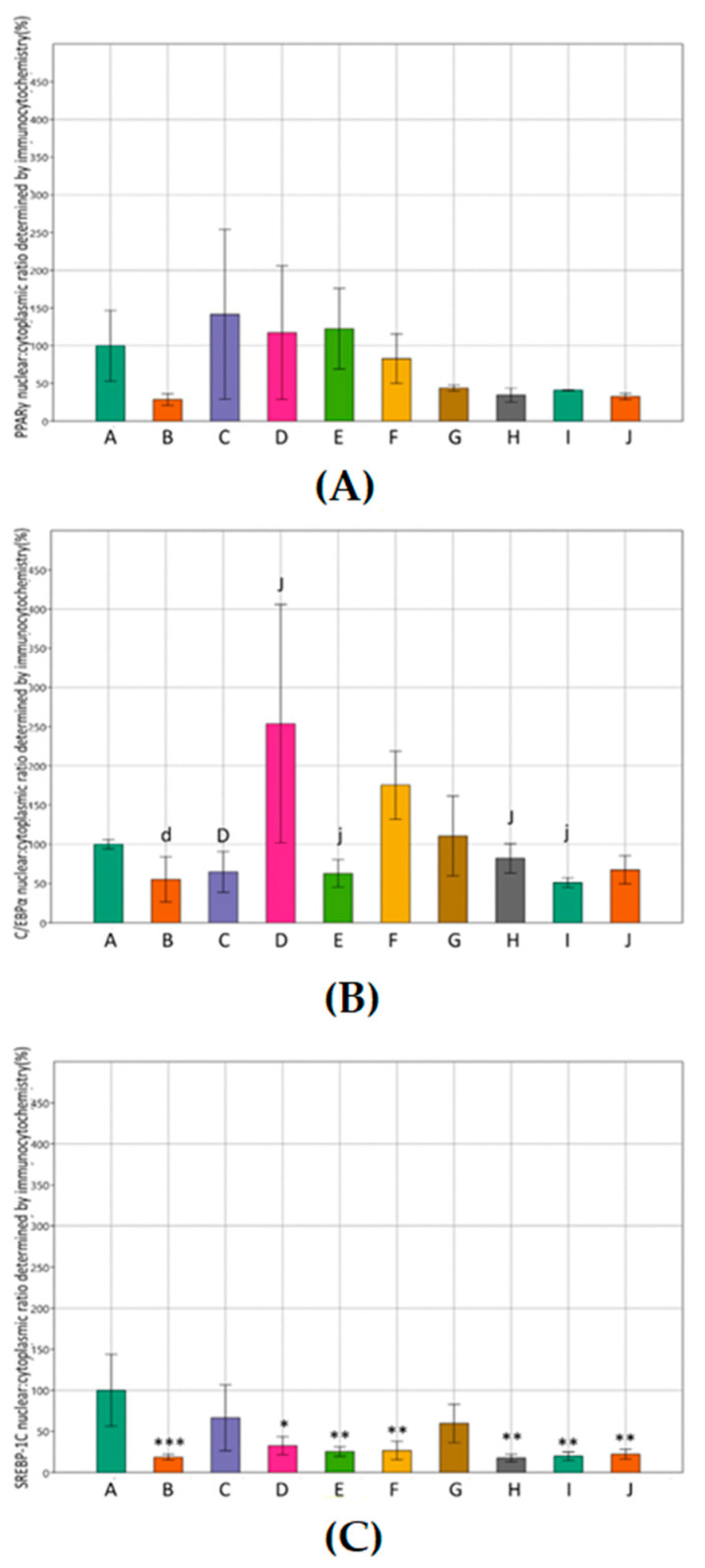
Effects of metformin and simvastatin on nuclear: cytoplasmic ratio of adipogenic proteins in 3T3-L1 cells, determined by immunocytochemistry. A = differentiated cells, B = cells treated with metformin 200 µM, C = cells treated with metformin 2 mM, D = C = cells treated with metformin 4 mM, E = cells treated with simvastatin 100 nM, F = cells treated with simvastatin 1 µM, G = cells treated with simvastatin 2 µM, H = cells treated with the combination of metformin 200 µM and simvastatin 100 nM, I = cells treated with the combination of metformin 2 mM and simvastatin 1 µM, J = cells treated with the combination of metformin 4 mM and simvastatin 2 µM, PPARγ = peroxisome proliferator-activated receptor γ, C/EBPα = CCAAT/enhancer binding protein α, SREBP-1C = sterol regulatory element-binding protein 1. The data are shown as the means ± SD (standard deviation) from three independent experiments. Data represents a percentage relative to differentiated cells as the control group. (**A**) Effects of metformin and simvastatin on nuclear: cytoplasmic ratio of PPARγ during the differentiation of 3T3-L1 cells (One-way ANOVA _F(9,26) = 2.006, *p* = 0.1034_ with Tukey HSD post hoc test); (**B**) effects of metformin and simvastatin on nuclear: cytoplasmic ratio of C/EBPα during the differentiation of 3T3-L1 cells (One-way ANOVA _F(9,31) = 3.993, *p* = 0.003855_ with Tukey HSD post hoc test; * *p* < 0.05, ** *p* < 0.01); (**C**) effects of metformin and simvastatin on nuclear: cytoplasmic ratio of SREBP-1C during the differentiation of 3T3-L1 cells (One-way ANOVA _F(9,34) = 5.666, *p* = 0.0002732_ with Tukey HSD post hoc test; * *p* < 0.05, ** *p* < 0.01, *** *p* < 0.001).

**Figure 6:**
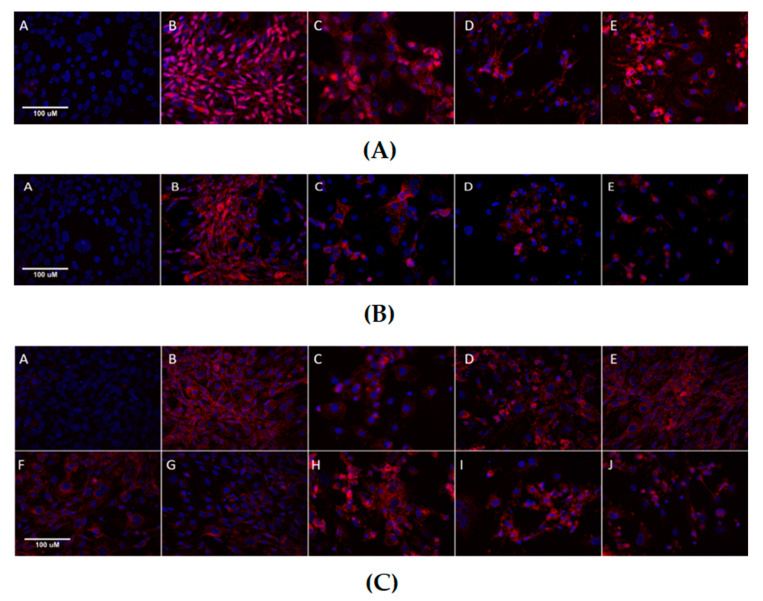
Representative microscopic images presenting immunofluorescence of adipogenic proteins in 3T3-L1 cells upon treatment with metformin and simvastatin, captured on the Axioskop 2 MOT microscope, 400×, scale 100 µM. (**A**) Immunofluorescence of peroxisome proliferator-activated receptor γ (PPARγ) in 3T3-L1 cells upon treatment with metformin and simvastatin. Blue = DAPI, cell nuclei; red = Cy5-PE, PPARγ. A = negative control, B = differentiated cells, C = cells treated with the combination of metformin 200 µM and simvastatin 100 nM, D = cells treated with the combination of metformin 2 mM and simvastatin 1 µM, E = cells treated with the combination of metformin 4 mM and simvastatin 2 µM; (**B**) Immunofluorescence of CCAAT/enhancer binding protein α (C/EBPα) in 3T3-L1 cells upon treatment with metformin and simvastatin. Blue = DAPI, cell nuclei; red = Cy5-PE, C/EBPα. A = negative control, B = differentiated cells, C = cells treated with the combination of metformin 200 µM and simvastatin 100 nM, D = cells treated with the combination of metformin 2 mM and simvastatin 1 µM, E = cells treated with the combination of metformin 4 mM and simvastatin 2 µM; (**C**) Immunofluorescence of SREBP-1C in 3T3-L1 cells upon treatment with metformin and simvastatin. Blue = DAPI, cell nuclei; red = Cy5-PE, SREBP-1C. A = negative control, B = differentiated cells, C = cells treated with metformin 200 µM, D = cells treated with metformin 4 mM, E = cells treated with simvastatin 100 nM, F = cells treated with simvastatin 1 µM, G = cells treated with simvastatin 2 µM, H = cells treated with the combination of metformin 200 µM and simvastatin 100 nM, I = cells treated with the combination of metformin 2 mM and simvastatin 1 µM, J = cells treated with the combination of metformin 4 mM and simvastatin 2 µM.

Since there was no significant difference in protein expression levels using immunostaining upon the treatment with MET and SIM compared to the control group, we examined their nuclear: cytoplasmic ratio, e.g., the nuclear–cytoplasmic translocation (Figure 5). However, decreased immunofluorescence is still visible after histochemical staining in treated cells compared to the control group for every examined epitope, as shown in Figure 6. Nuclear: cytoplasmic ratio of PPARγ was not significantly affected with treatment (Figure 5A), while, for SREBP-1C, it was decreased in every treated group (Figure 5C). C/EBPα nuclear: cytoplasmic ratio was also decreased in most treated groups (Figure 5B).

## 4. Discussion

Adipose tissue plays an important role in the development of obesity and related diseases due to changes in adipocyte function and adipocytokine secretion. The formation of fat cells from preadipocytes includes morphological changes, an increase in cell number and size, the expression of lipogenic enzymes, and the accumulation of lipid droplets [22]. In this study, we aimed to use the existing knowledge and a defined adipogenesis model of 3T3-L1 cell line to detect molecular changes resulting from MET and SIM treatment. In particular, modulation of C/EBPα, PPARγ, and SREBP-1C expression, and accumulation of lipid droplets in differentiated adipocytes were analyzed.

An early response to the differentiation medium presents through the expression of the adipogenic genes, increasing glucose uptake and triglyceride synthesis [23]. Adipocyte differentiation depends on the coordinated regulation of gene expression in a complex transcription cascade necessary for the formation and maintenance of the adipocyte phenotype. A transient increase in the expression of C/EBPβ and δ at the beginning of the adipogenesis stimulates the transcription of C/EBPα and PPARγ, key transcription factors in adipogenesis [24,25]. PPARγ and C/EBPα, through positive feedback, promote the differentiation and induction of leptin, adiponectin, lipoprotein lipase, adipocyte protein 2, fatty acid synthase, perilipin, and, in the final stage of differentiation [26].

The effects of MET have already been investigated on 3T3-L1 cells, and the inhibition of adipogenesis by the addition of MET has been confirmed in several studies [2,27,28]. However, most of these studies used high doses of MET. Maximum serum MET levels in patients treated with 500–3000 mg/day are approximately 20 µM; therefore, lower MET concentrations in in vitro studies should be investigated. In our study, exposing the cells to the adipogenic cocktail increased lipid accumulation, which was decreased with MET and SIM treatment, but this was not statistically significant. In a study by Chen et al., where cells treated with lower doses of MET (1.25 mM, 2.5 mM) showed increased intensity of Oli Red O staining, but higher concentrations (5 mM, 10 mM) reduced the intensity of staining compared to control cells [2]. In addition, Alexandre et al. in their study examined the effect of 2–16 mM MET on adipogenesis in 3T3-L1 cells, where 2 mM showed no inhibitory but mildly stimulating effect on adipogenesis, while higher concentrations had inhibitory effects [27]. As for the statins, most of the in vitro studies assessed the effect of atorvastatin, which was shown to be effective in inhibiting adipogenesis at the doses equivalent to the plasma concentrations in statin-treated patients [29]. The difference in the mechanism of action between atorvastatin and SIM has not yet been elucidated, and the effective concentration of SIM used in cell culture was higher than the plasma concentrations of this drug [30]. In our study, the inhibition of lipid accumulation was observed at all concentrations of SIM, and previous reports also achieved inhibition at 1 µM [31,32]. However, in one study, this effect was observed between the third and fifth day of differentiation, suggesting that time period in which treatment exerts its effects is important. In our study, the highest concentration of combined MET-SIM treatment showed reduction in lipid accumulation regardless of the dose, which was not statistically significant but distinctively visible in Oil Red O staining.

The previously mentioned study by Chen et al. showed that a lower dose of MET (1.25 mM) induced, while higher doses (5 mM) inhibited, adipogenic genes (PPARγ, C/EBPα, SREBP-1C), which was further confirmed for PPARγ and C/EBPα protein expression [2]. In our study, MET down-regulated the expression of adipogenic markers. Similar results can be found in several other studies that showed lower mRNA and protein levels of PPARγ and C/EBPα in MET-treated cells up to 5 mM [33,34,35,36], and decreased expression of SREBP-1 [36]. Statins were shown to suppress adipogenesis by reducing the expression of PPARγ, C/EBPα, SREBP-1C, and leptin and adiponectin [37]. In our study, SIM down-regulated adipogenic transcription factors, with the one exception for mRNA levels of C/EBPα in cells treated with 100 nM, which was, however, not accompanied by lower protein levels of C/EBPα. These results also confirmed previously reported effects of SIM on decreasing C/EBPα mRNA expression at 1 μM, consequently suppressing leptin secretion [38]. The most prominent results were achieved with combined MET-SIM treatment that significantly decreased mRNA and protein levels of PPARγ, C/EBPα, and SREBP-1C at the highest dose. One comparable study on effects of MET and SIM showed that atorvastatin (up to 20 µM) decreased mRNA expression of PPARγ and C/EBPα in 3R3-L1 cell culture, which was enhanced in combination with MET [18]. However, one finding was particularly interesting, and that is a greater effect of SIM alone on the protein expression of PPARγ than the effect of the combined treatment. This could be due to pleiotropic effects of statins that can arise from modulation of membrane spanning proteins through changes in protein prenylation, cholesterol levels, and changes in lipid bilayer properties, thereby altering membrane protein function [39]. This could affect the transition of proteins and nucleic acids through cell membrane, which can be a possible explanation to SIM being more effective than combined treatment.

In addition to these findings, we have examined the nuclear: cytoplasmic ratio of PPARγ, C/EBPα, and SREBP-1C upon MET and SIM treatment. Considering that transcription factors reside primarily in the nucleus, we aimed to see if the treatment would affect nuclear import/export pathways, causing the nuclear–cytoplasmic translocation [40]. In our study, combined MET-SIM treatment significantly reduced nuclear: cytoplasmic ratio of C/EBPα and SREBP-1C. This result could lead us to conclusion that the treatment decreased not only protein expression but also affected their subcellular localization, which should definitely be the subject of further extensive studies.

There are still several matters to attend to when discussing in vitro studies as models for different diseases. First, cell cultures differentiate almost exclusively into white adipocyte adipocytes, while, in humans, there is also brown adipose tissue responsible primarily for energy expenditure. Furthermore, the rodent cell line cannot fully reflect the effect of MET+SIM in humans. Another obstacle is that the cell line does not allow research into the behavior of different adipose tissue stores, which exist in different locations in humans and each has a different adipogenic potential. Differences in the metabolic behavior of mature fat cells within different adipose tissue stores can have significant clinical consequences, most notably in the difference between subcutaneous and visceral adipose tissue, where the latter is a higher risk factor for insulin resistance and dyslipidemia. Adipose tissue is also dependent on gender and the complex effect of hormones, which cannot be accurately simulated in the laboratory conditions. One of the potential disadvantages of in vitro statin efficacy studies is the use of higher doses than their plasma concentrations in patients. Most studies use statin concentrations of 1 to 50 μM, while the mean serum statin concentration of patients on therapeutic doses is 1 to 15 nM [41]. Peak statin concentrations are 2–6 times higher than mean concentrations, although they are present in serum for only a few minutes, while exposure to statin in cell cultures is measured in days.

In the present study, we showed that combined MET and SIM treatment have the inhibitory effects on adipogenesis and lipid accumulation in 3T3-L1 adipocytes by decreasing mRNA and protein expression of PPARγ, C/EBPα, and SREBP-1C. These findings might be beneficial to the development of treatment strategies for obesity and obesity-related disorders in the future.

## Data Availability

The data presented in this study are available in the supplementary material of this manuscript.

## Supplementary Materials

The following are available online at https://www.mdpi.com/article/10.3390/cimb43030144/s1.
