# Supplementary figures and images for: Effect of Metformin and Simvastatin in Inhibiting Proadipogenic Transcription Factors
Source: Curr Issues Mol Biol. 2021 Nov 25;43(3):2082–97. doi: 10.3390/cimb43030144 (PMC8929042; doi:10.3390/cimb43030144)

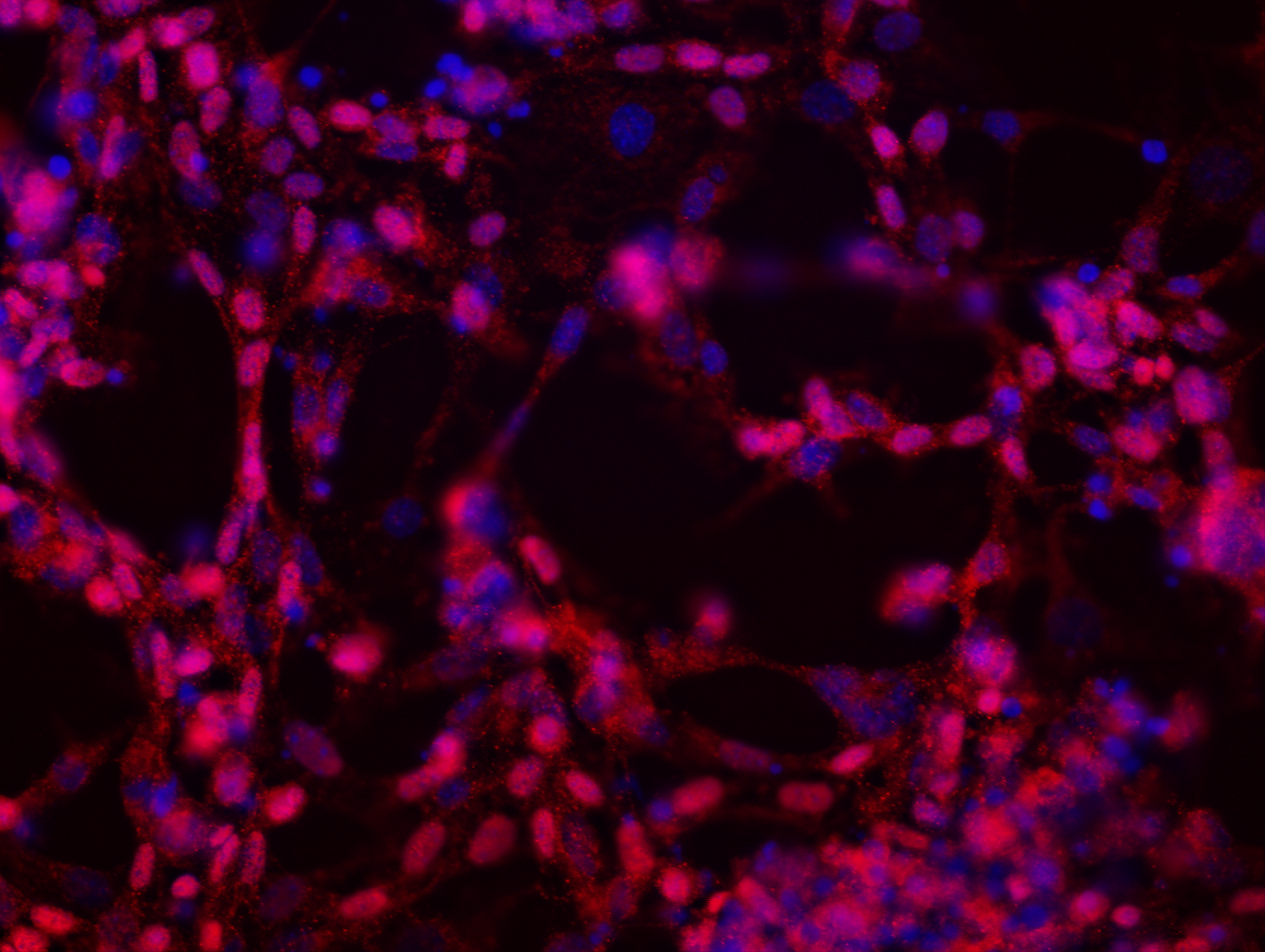

Supplement: Supplementary file 1 [file cimb-43-00144-s001.zip › cimb-1454926-supplementary/Ppar/Differentiated/1.tif]

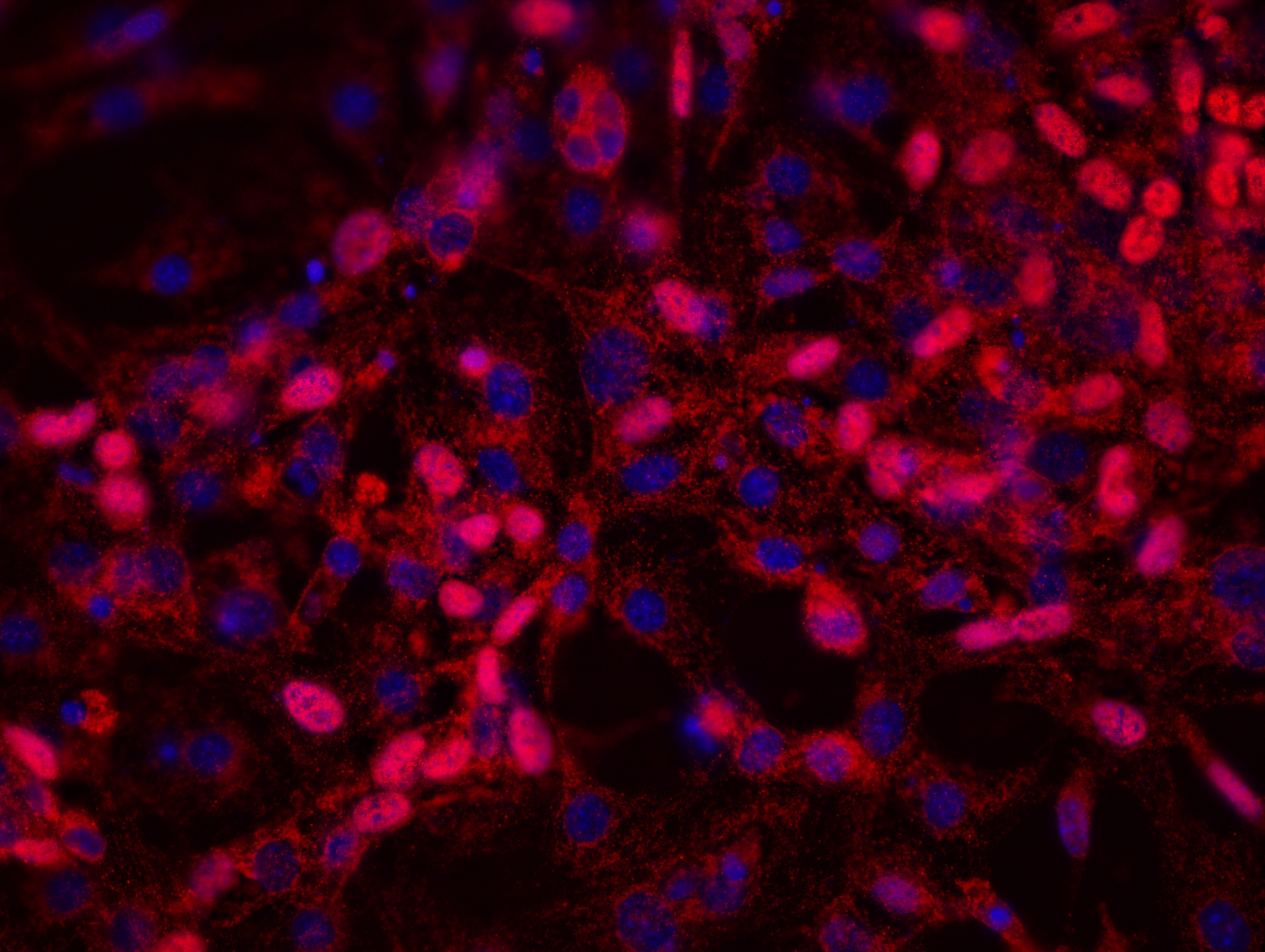

Supplement: Supplementary file 1 [file cimb-43-00144-s001.zip › cimb-1454926-supplementary/Ppar/Differentiated/2.tif]

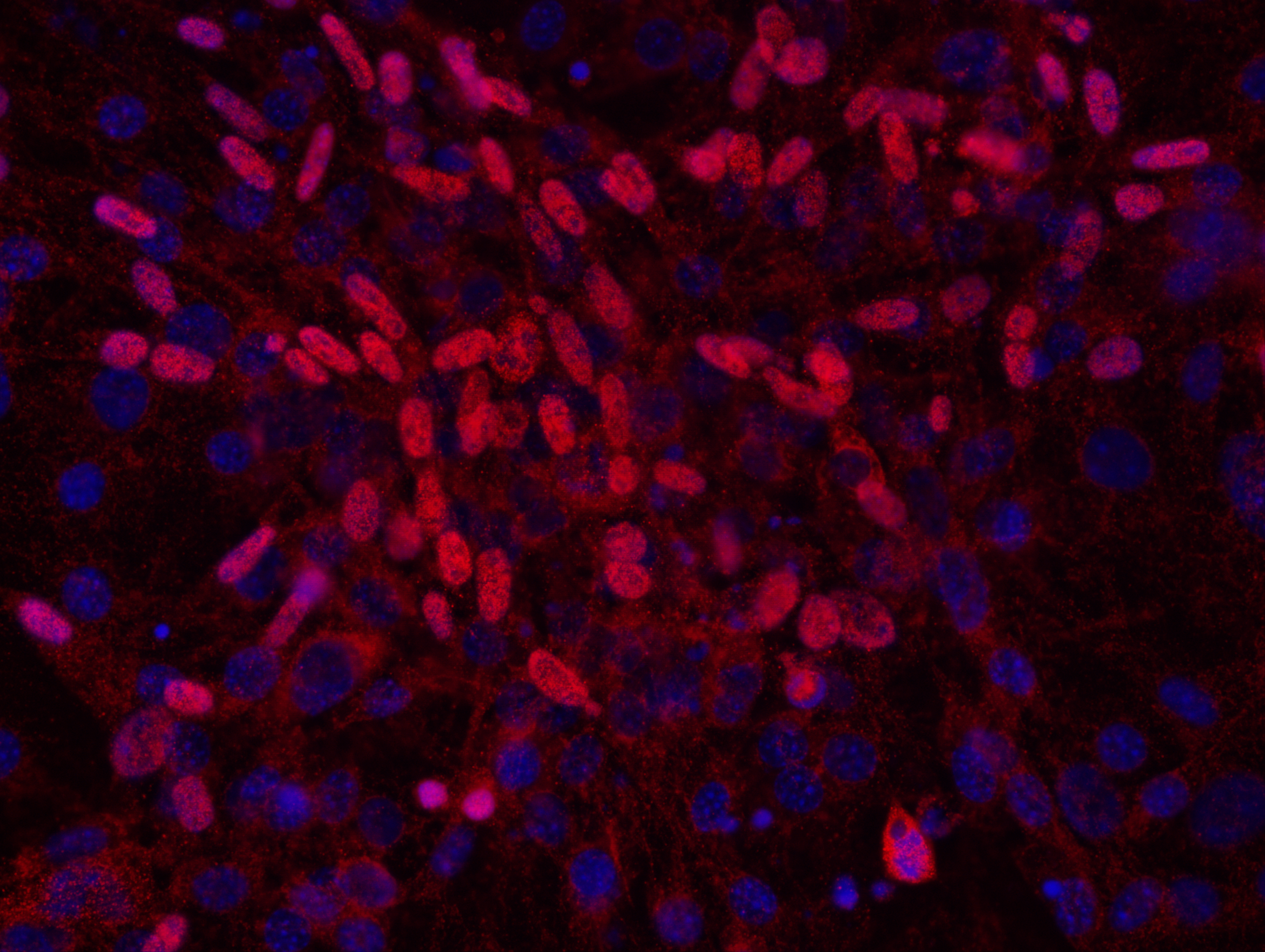

Supplement: Supplementary file 1 [file cimb-43-00144-s001.zip › cimb-1454926-supplementary/Ppar/Differentiated/3.tif]

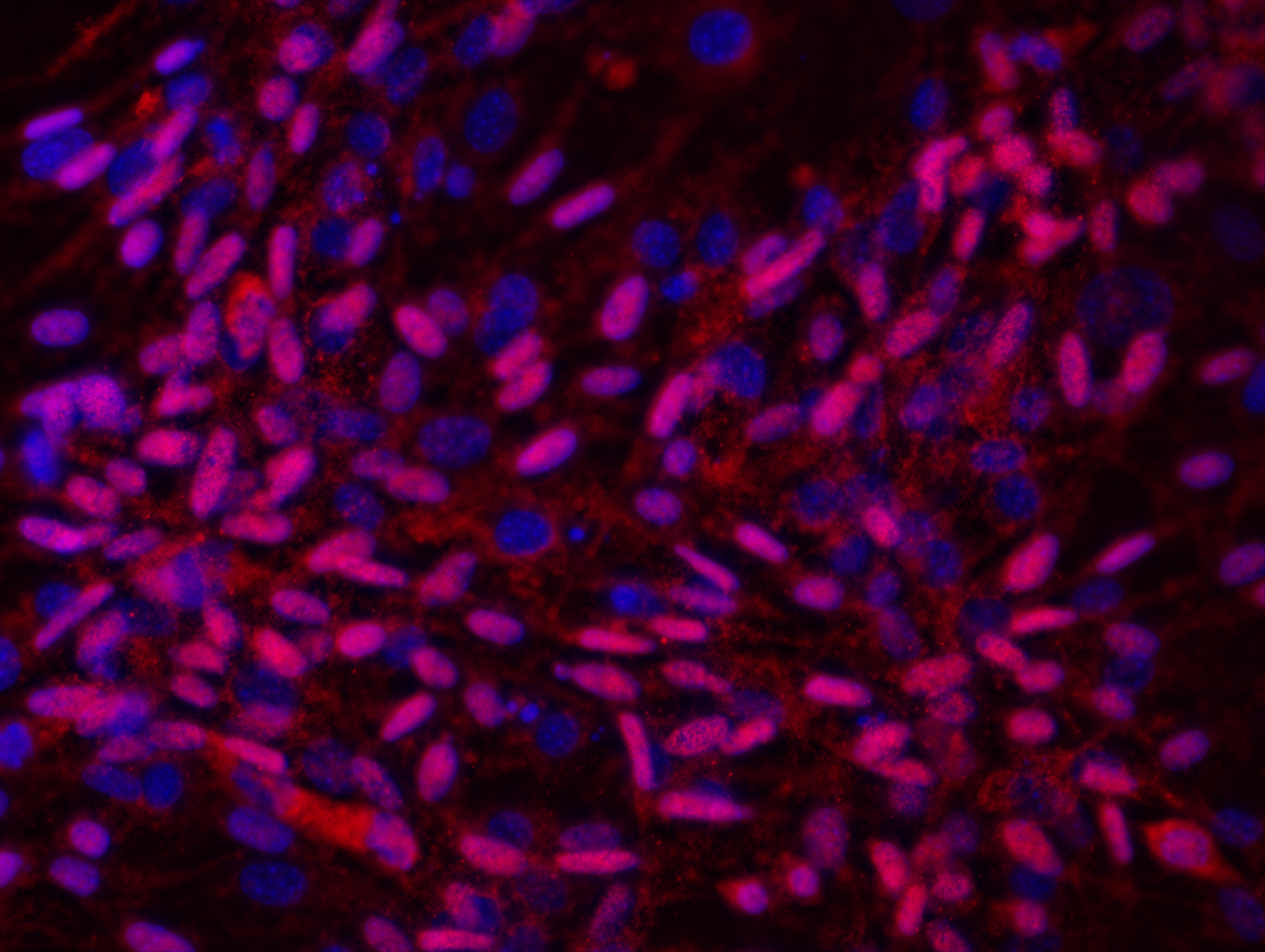

Supplement: Supplementary file 1 [file cimb-43-00144-s001.zip › cimb-1454926-supplementary/Ppar/Differentiated/4.tif]

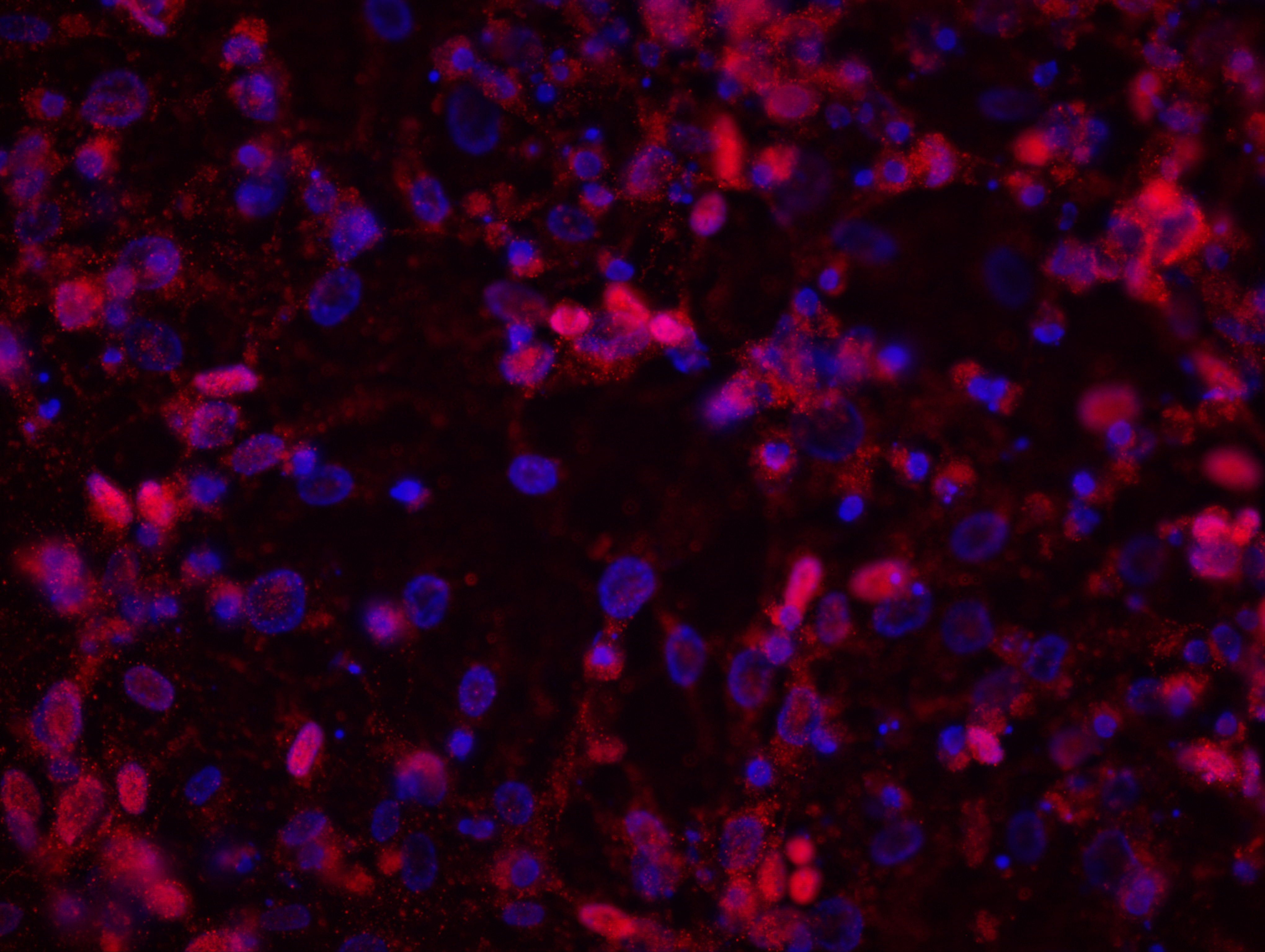

Supplement: Supplementary file 1 [file cimb-43-00144-s001.zip › cimb-1454926-supplementary/Ppar/M1/1.tif]

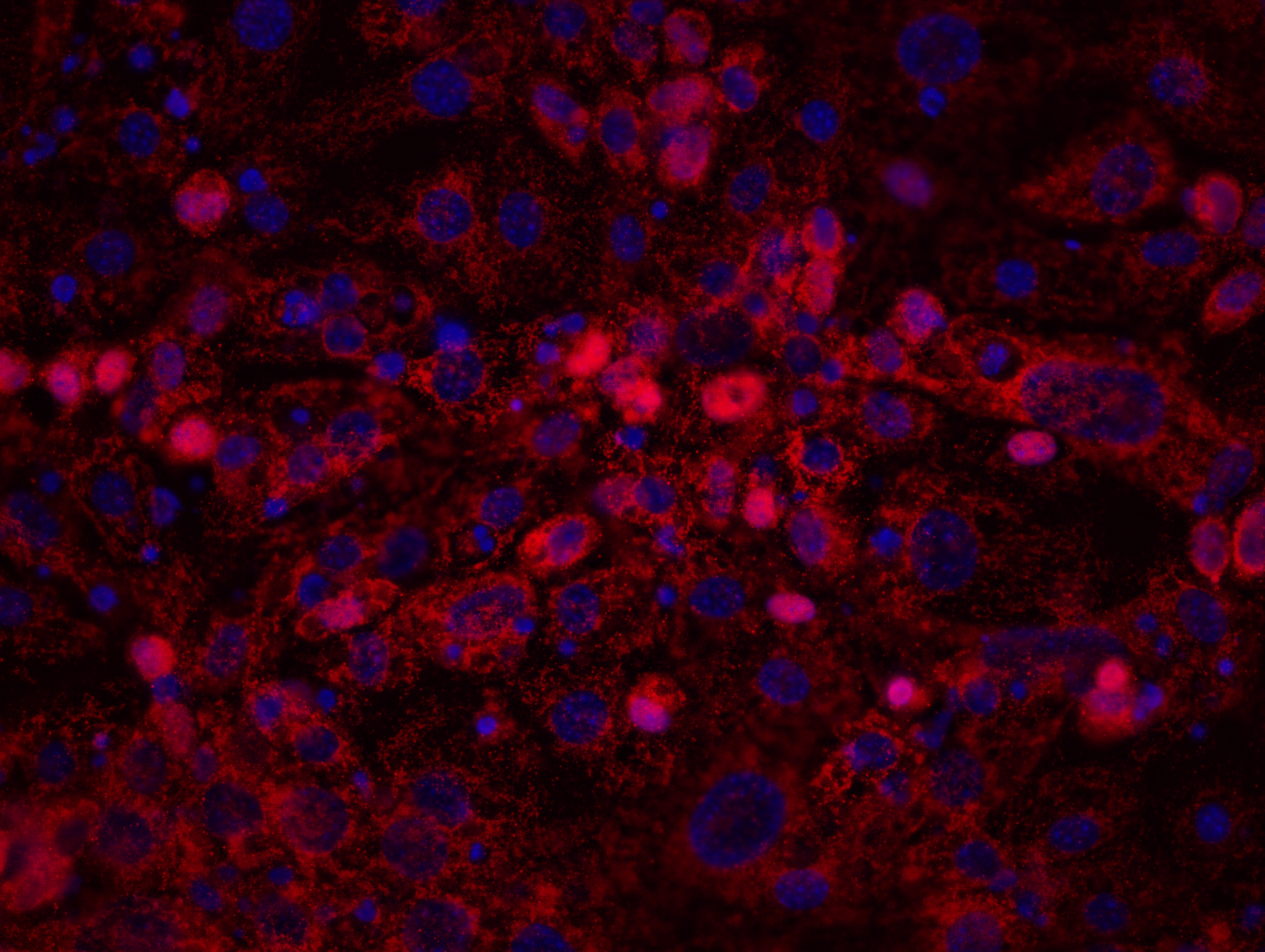

Supplement: Supplementary file 1 [file cimb-43-00144-s001.zip › cimb-1454926-supplementary/Ppar/M1/2.tif]

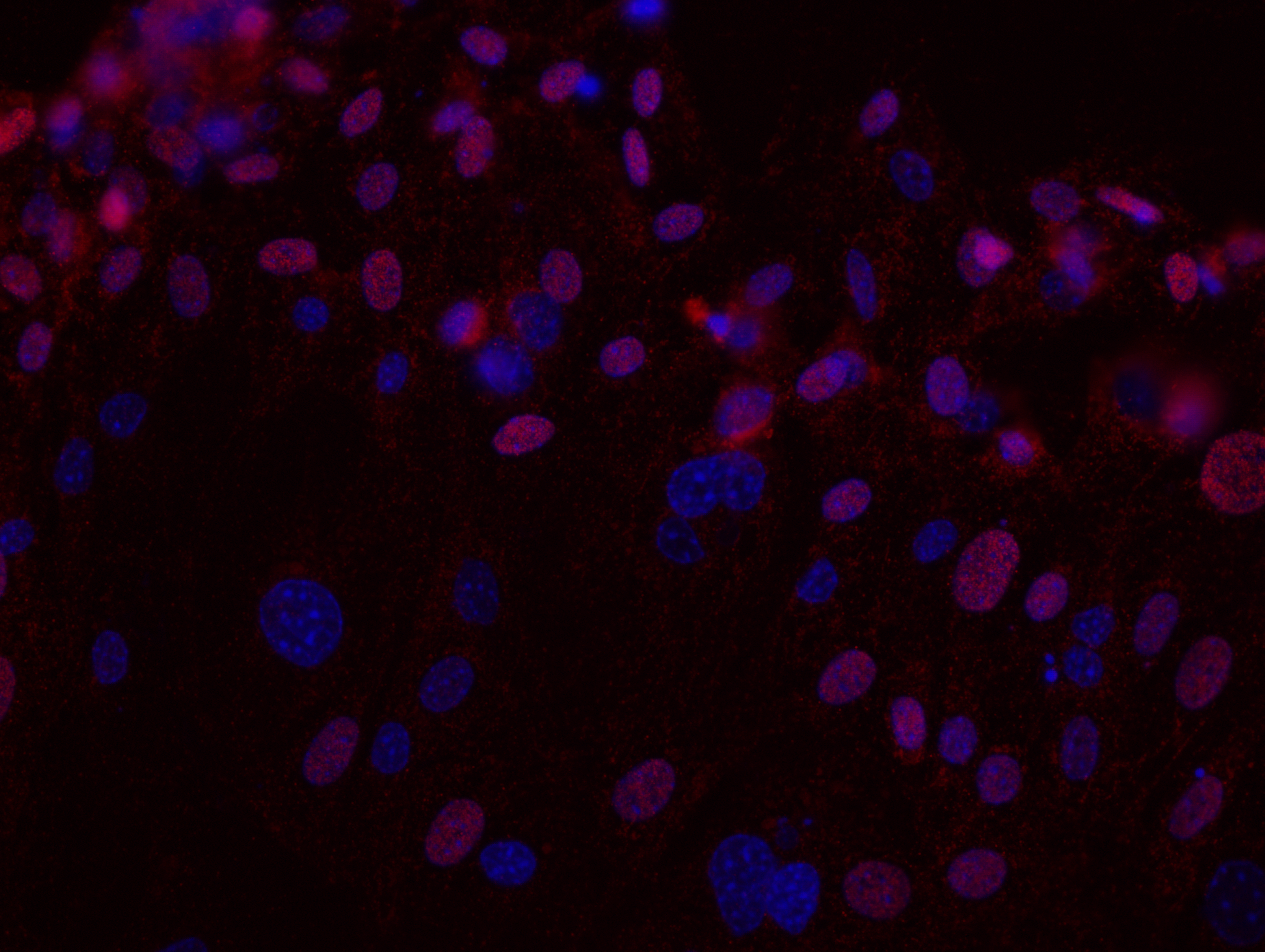

Supplement: Supplementary file 1 [file cimb-43-00144-s001.zip › cimb-1454926-supplementary/Ppar/M1/3.tif]

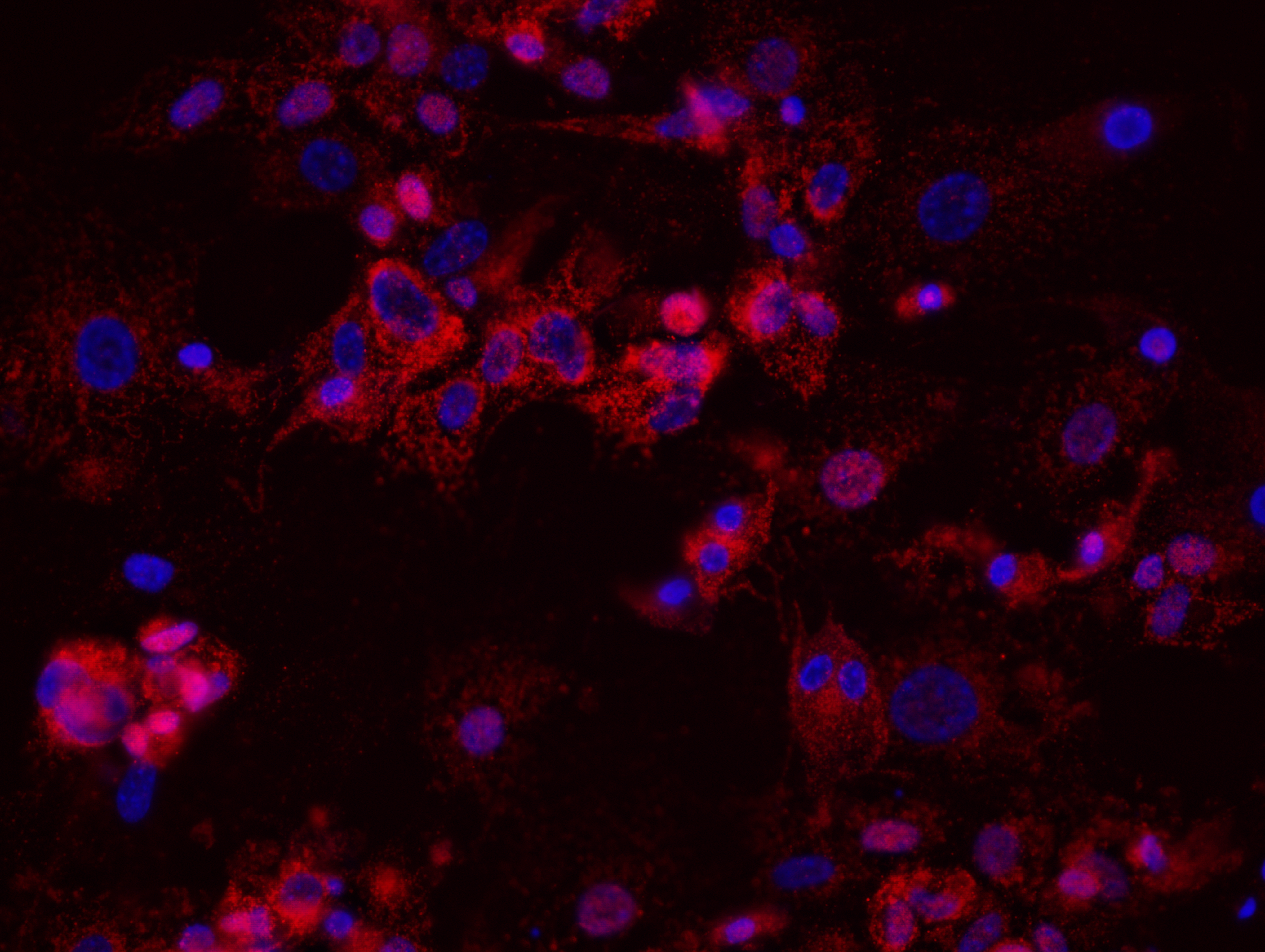

Supplement: Supplementary file 1 [file cimb-43-00144-s001.zip › cimb-1454926-supplementary/Ppar/M1/4.tif]

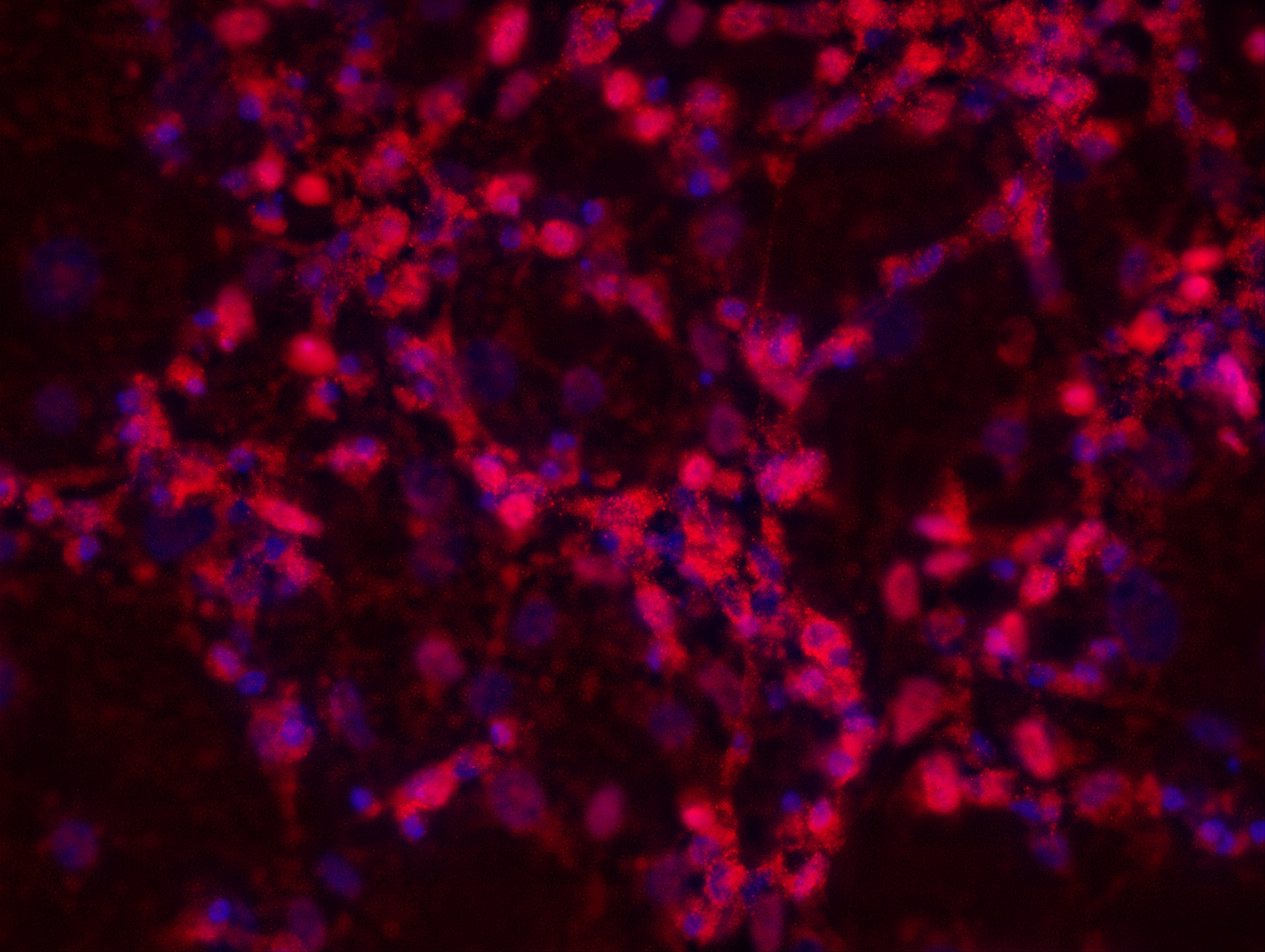

Supplement: Supplementary file 1 [file cimb-43-00144-s001.zip › cimb-1454926-supplementary/Ppar/M2/1.tif]

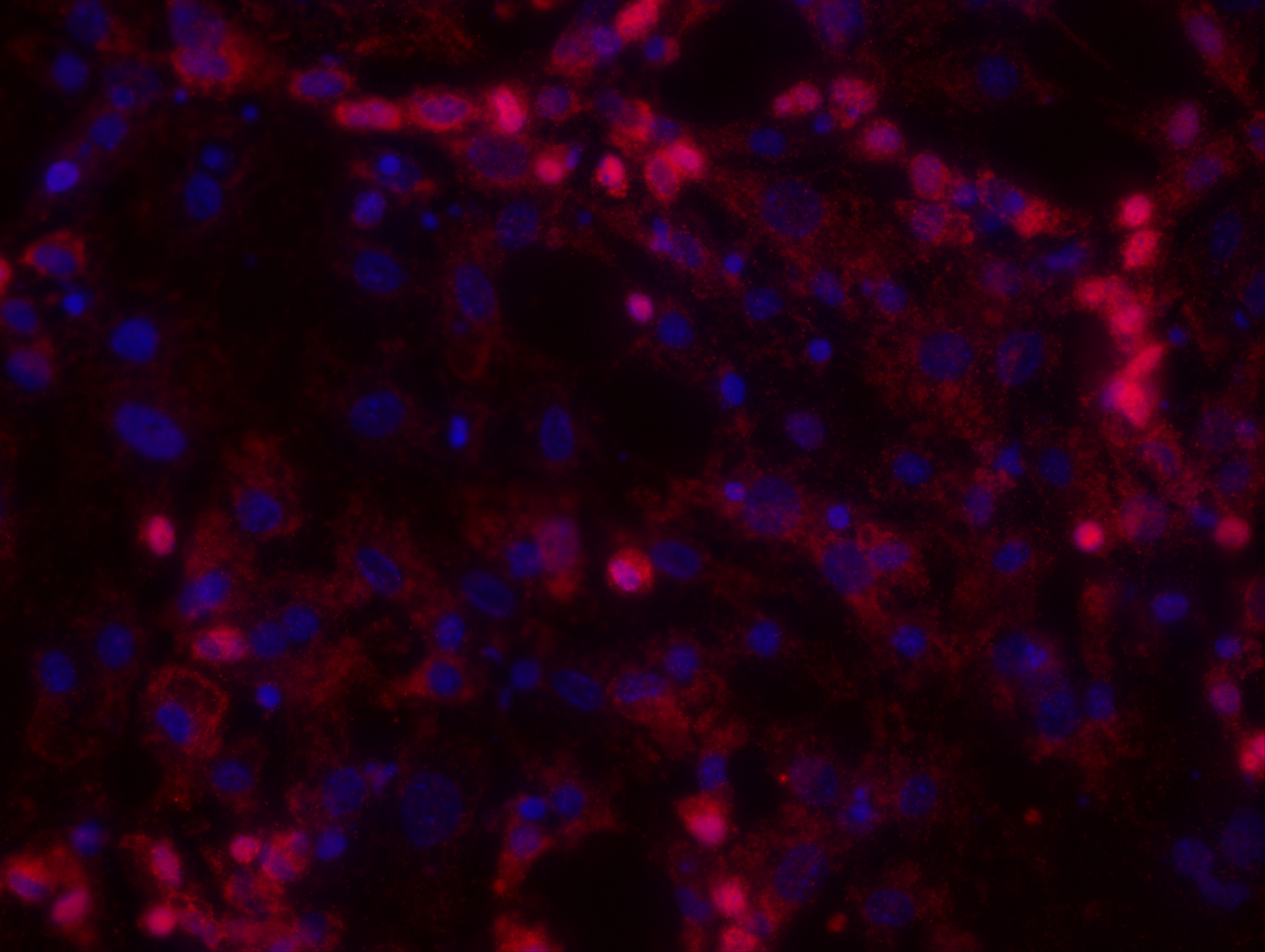

Supplement: Supplementary file 1 [file cimb-43-00144-s001.zip › cimb-1454926-supplementary/Ppar/M2/2.tif]

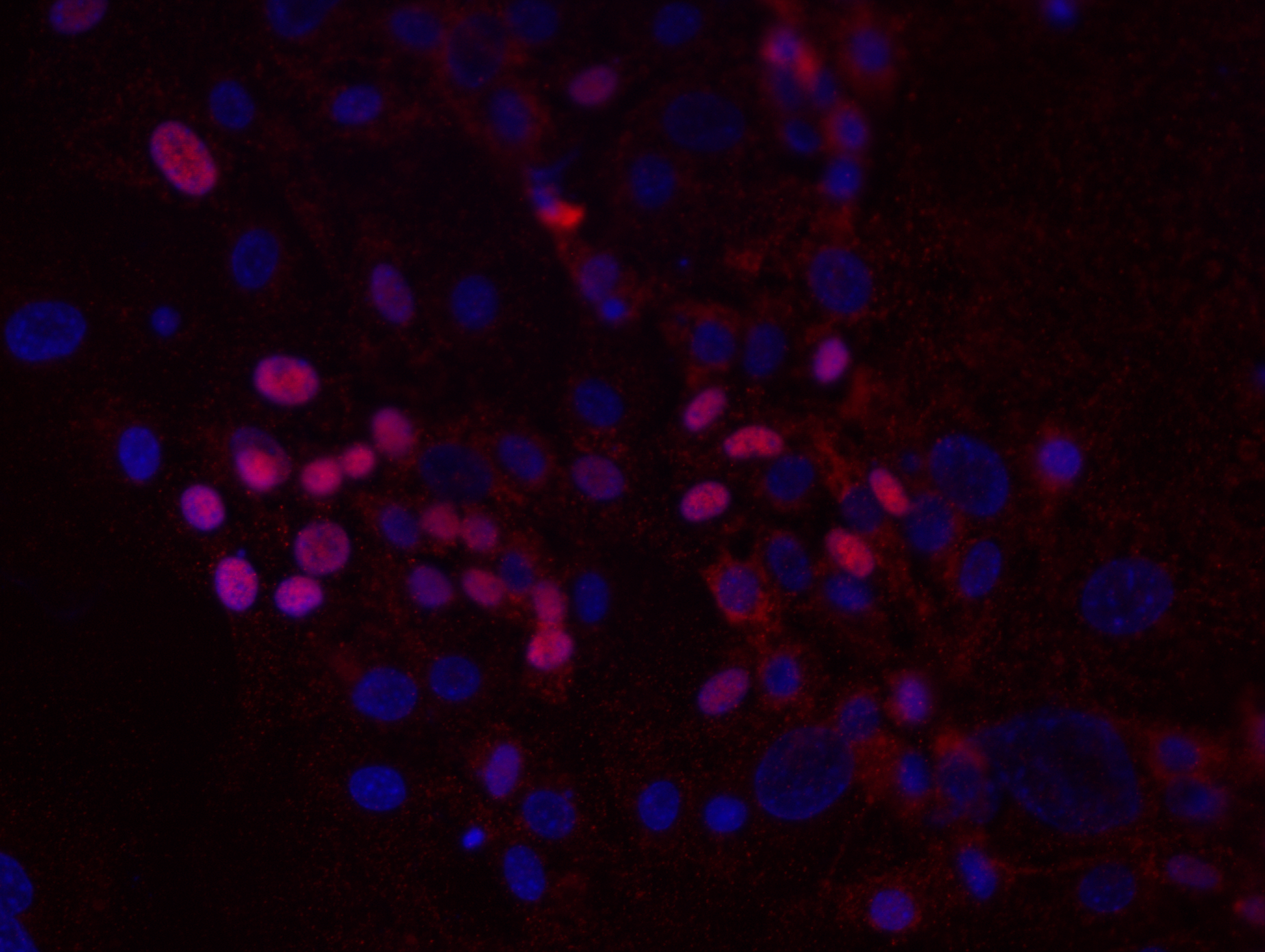

Supplement: Supplementary file 1 [file cimb-43-00144-s001.zip › cimb-1454926-supplementary/Ppar/M2/3.tif]

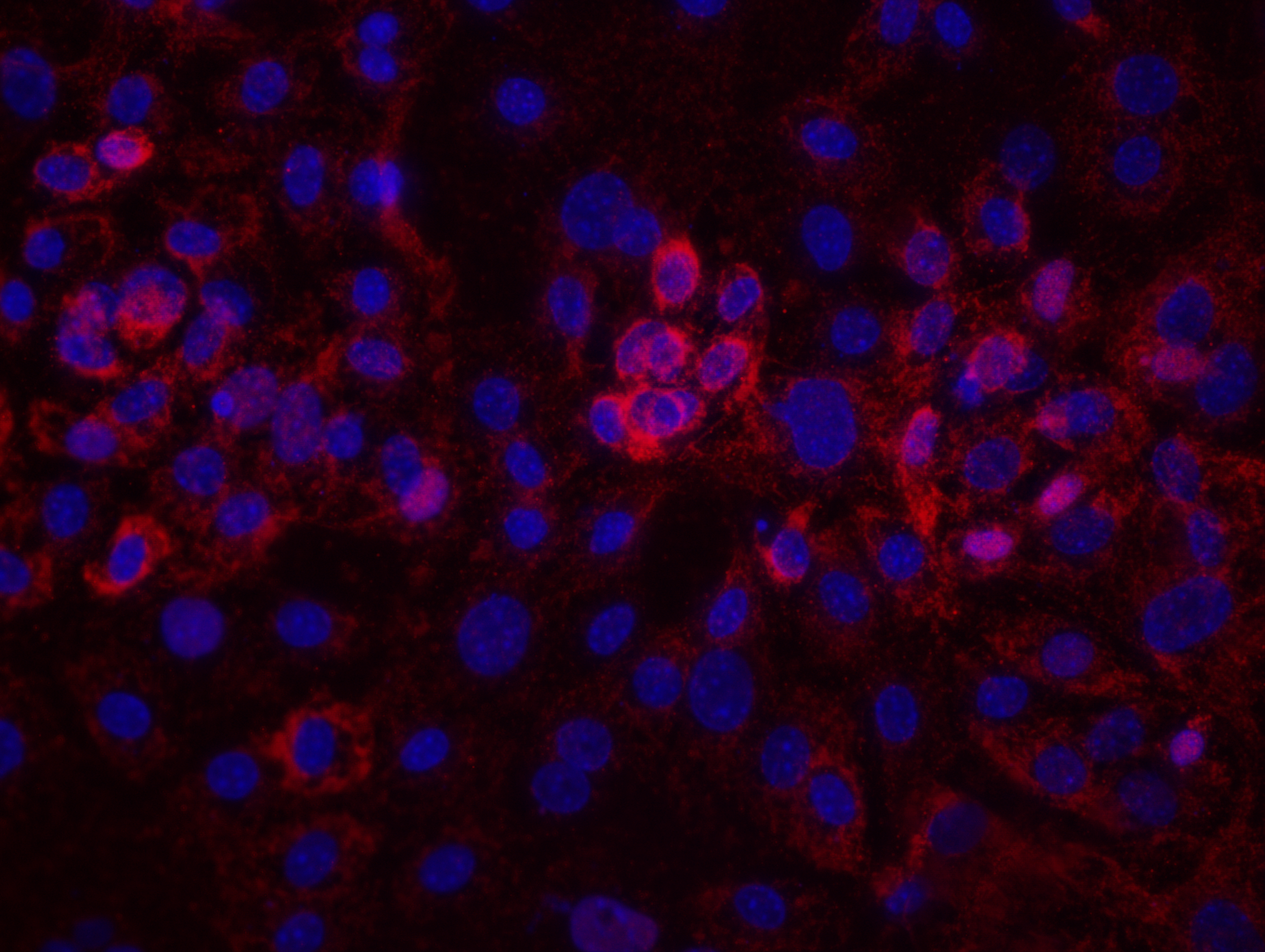

Supplement: Supplementary file 1 [file cimb-43-00144-s001.zip › cimb-1454926-supplementary/Ppar/M2/4.tif]

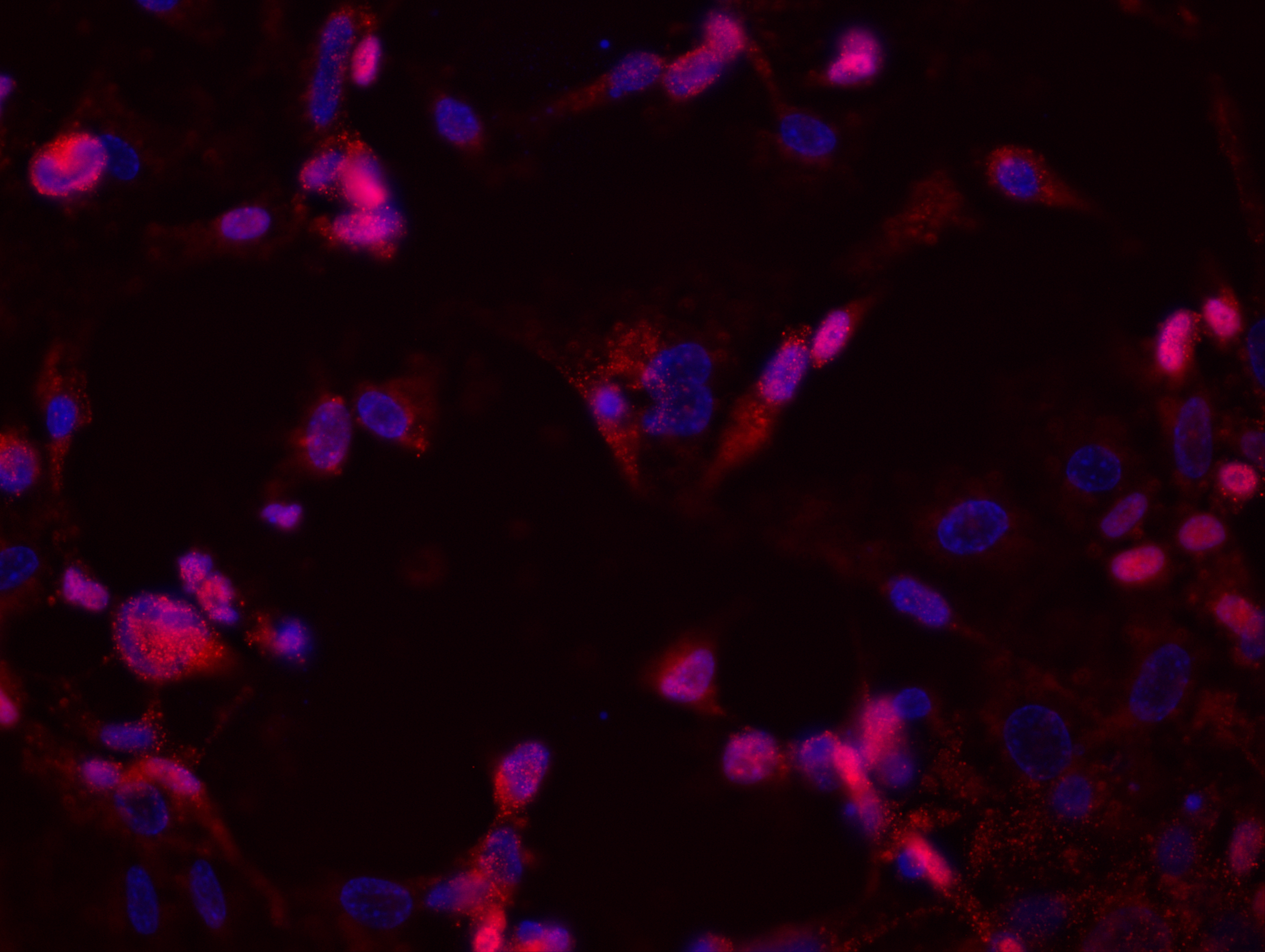

Supplement: Supplementary file 1 [file cimb-43-00144-s001.zip › cimb-1454926-supplementary/Ppar/M3/1.tif]

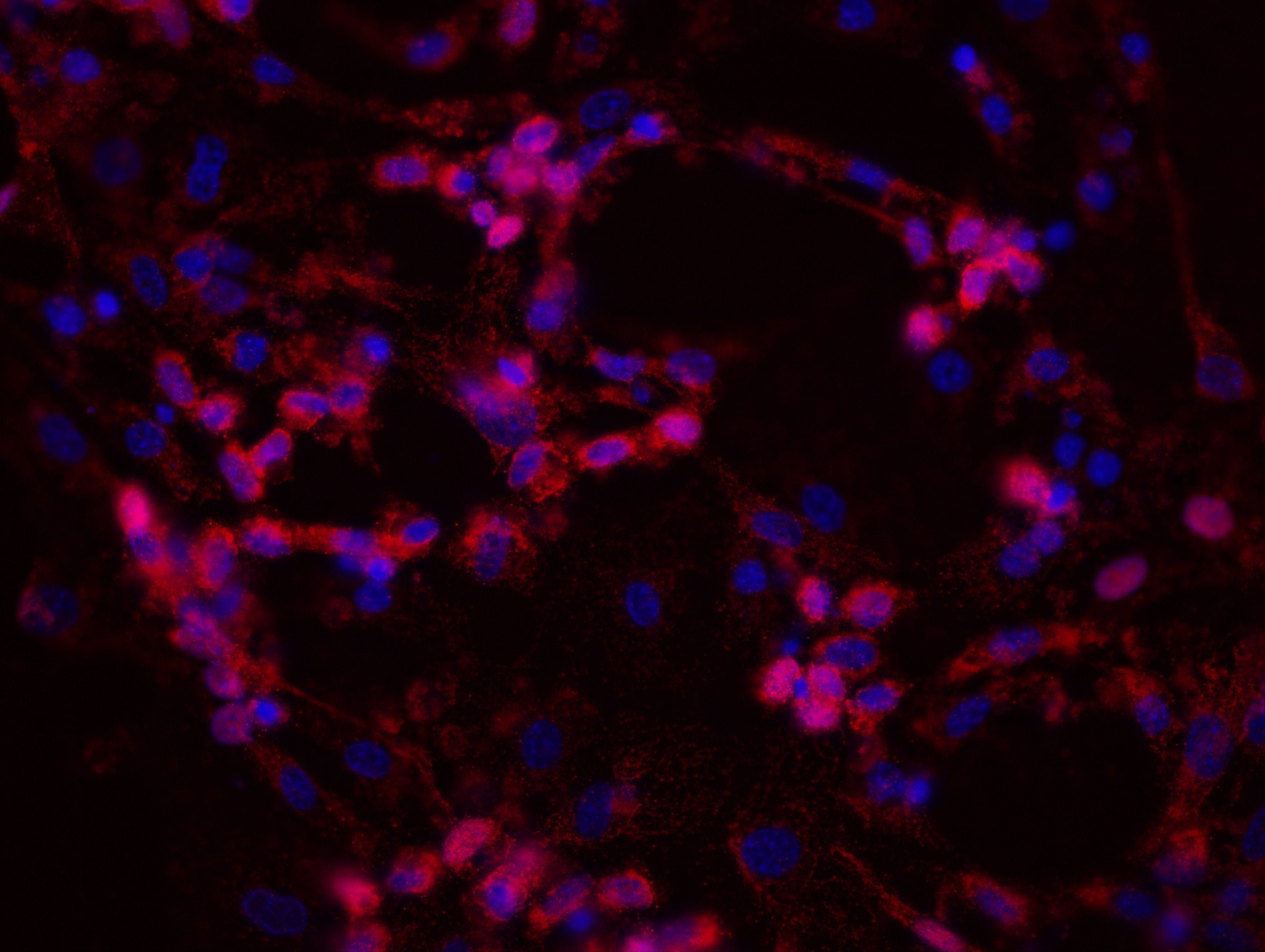

Supplement: Supplementary file 1 [file cimb-43-00144-s001.zip › cimb-1454926-supplementary/Ppar/M3/2.tif]

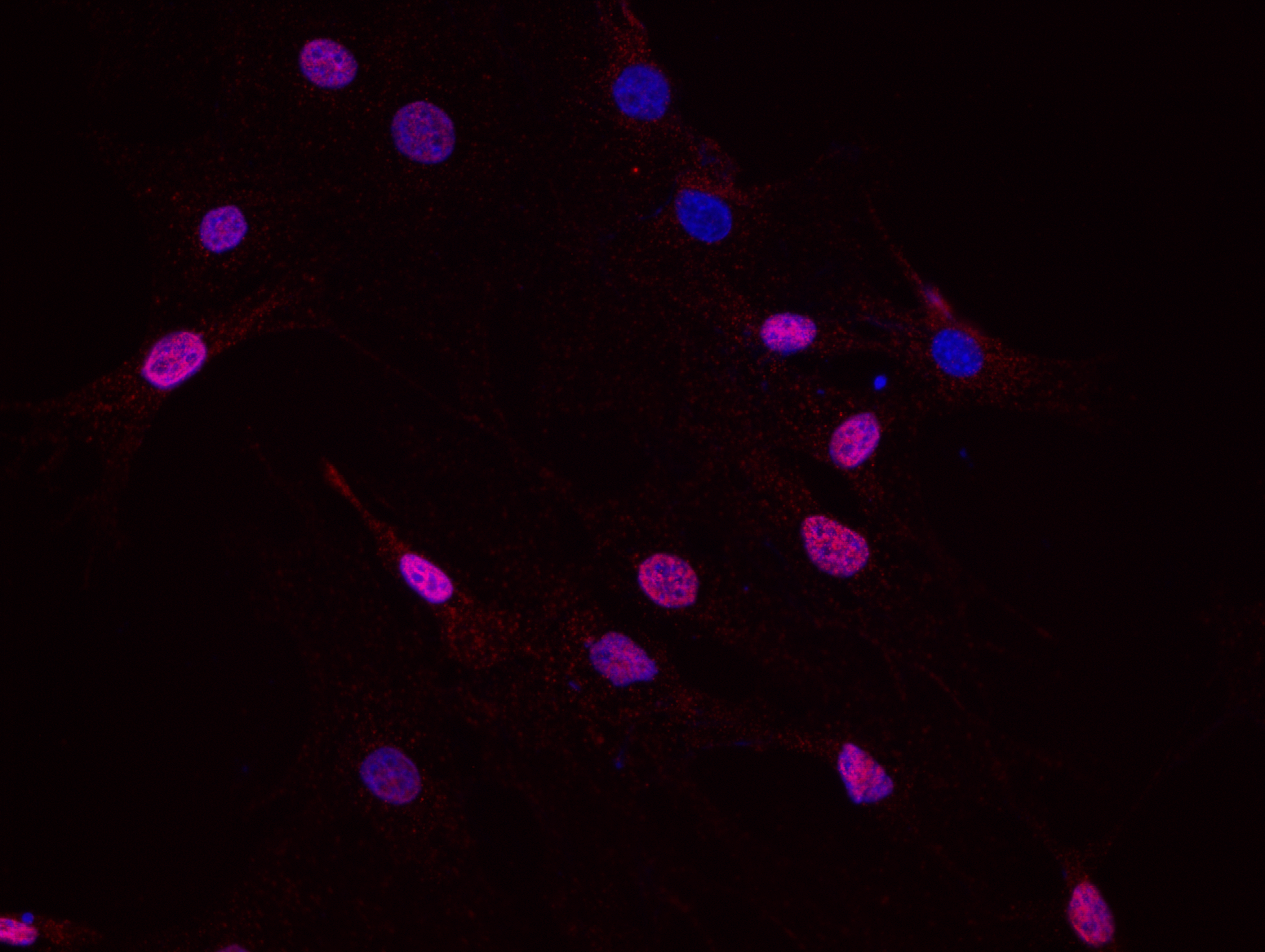

Supplement: Supplementary file 1 [file cimb-43-00144-s001.zip › cimb-1454926-supplementary/Ppar/M3/3.tif]

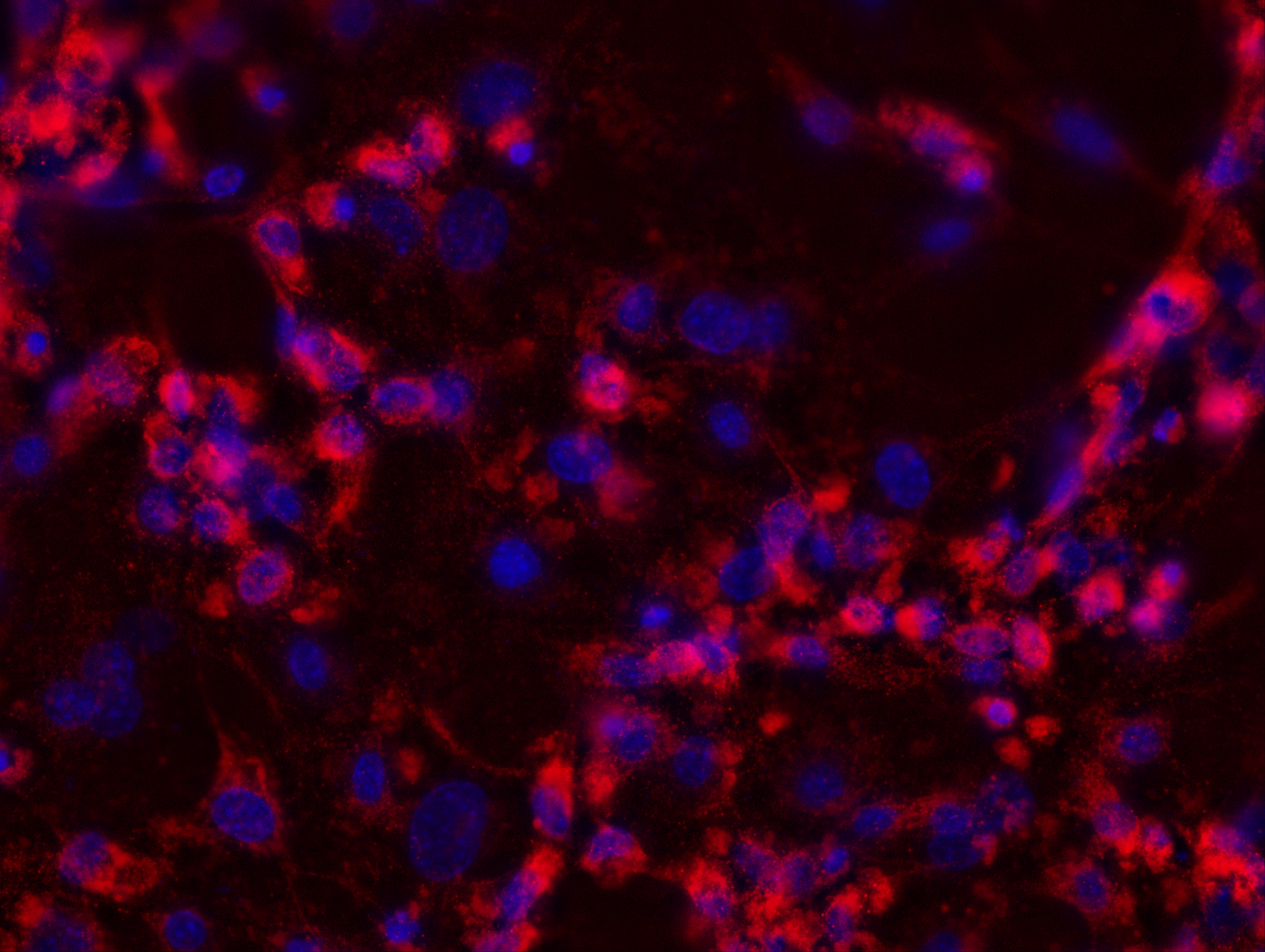

Supplement: Supplementary file 1 [file cimb-43-00144-s001.zip › cimb-1454926-supplementary/Ppar/M3/4.tif]

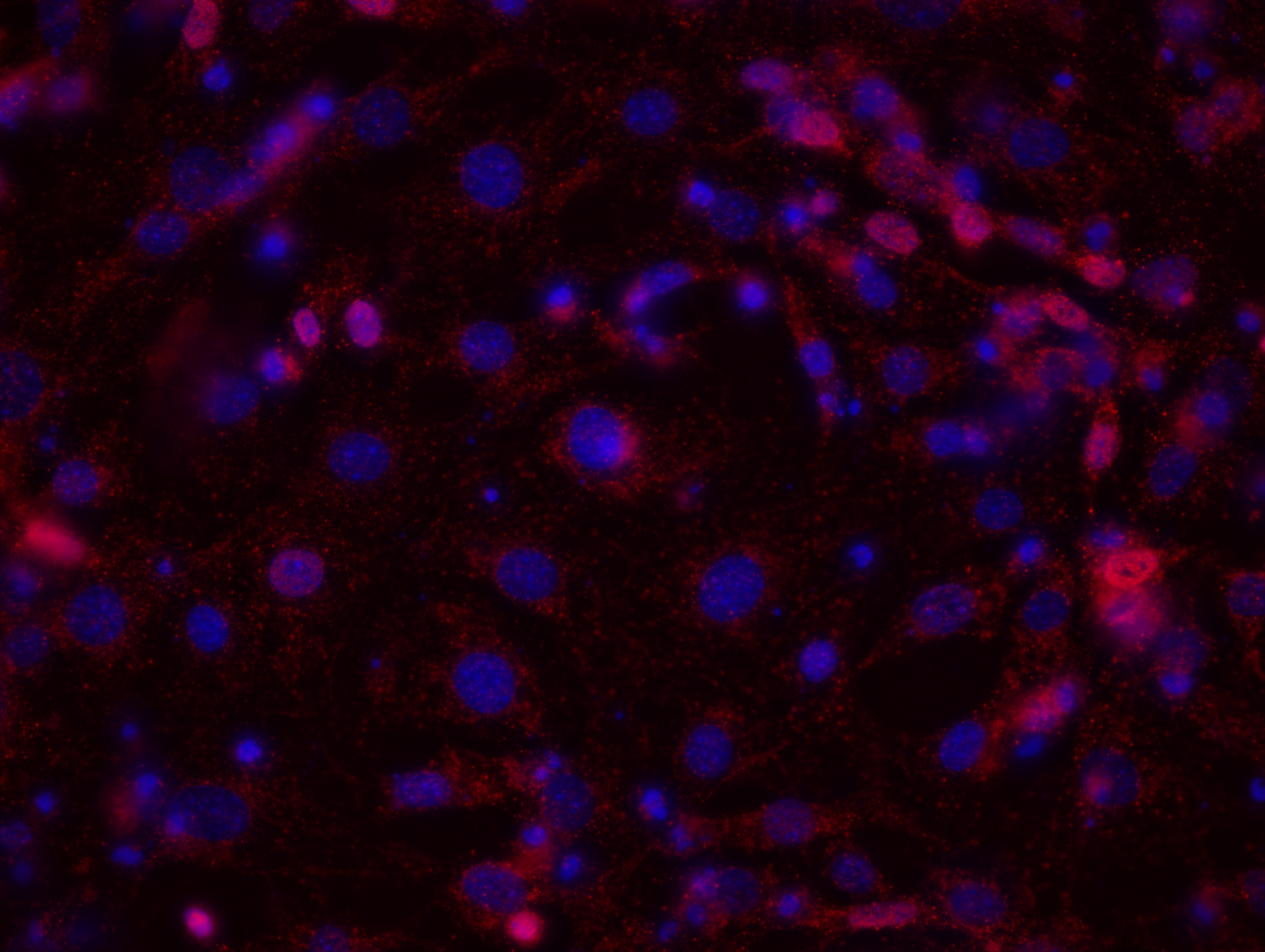

Supplement: Supplementary file 1 [file cimb-43-00144-s001.zip › cimb-1454926-supplementary/Ppar/S1/1.tif]

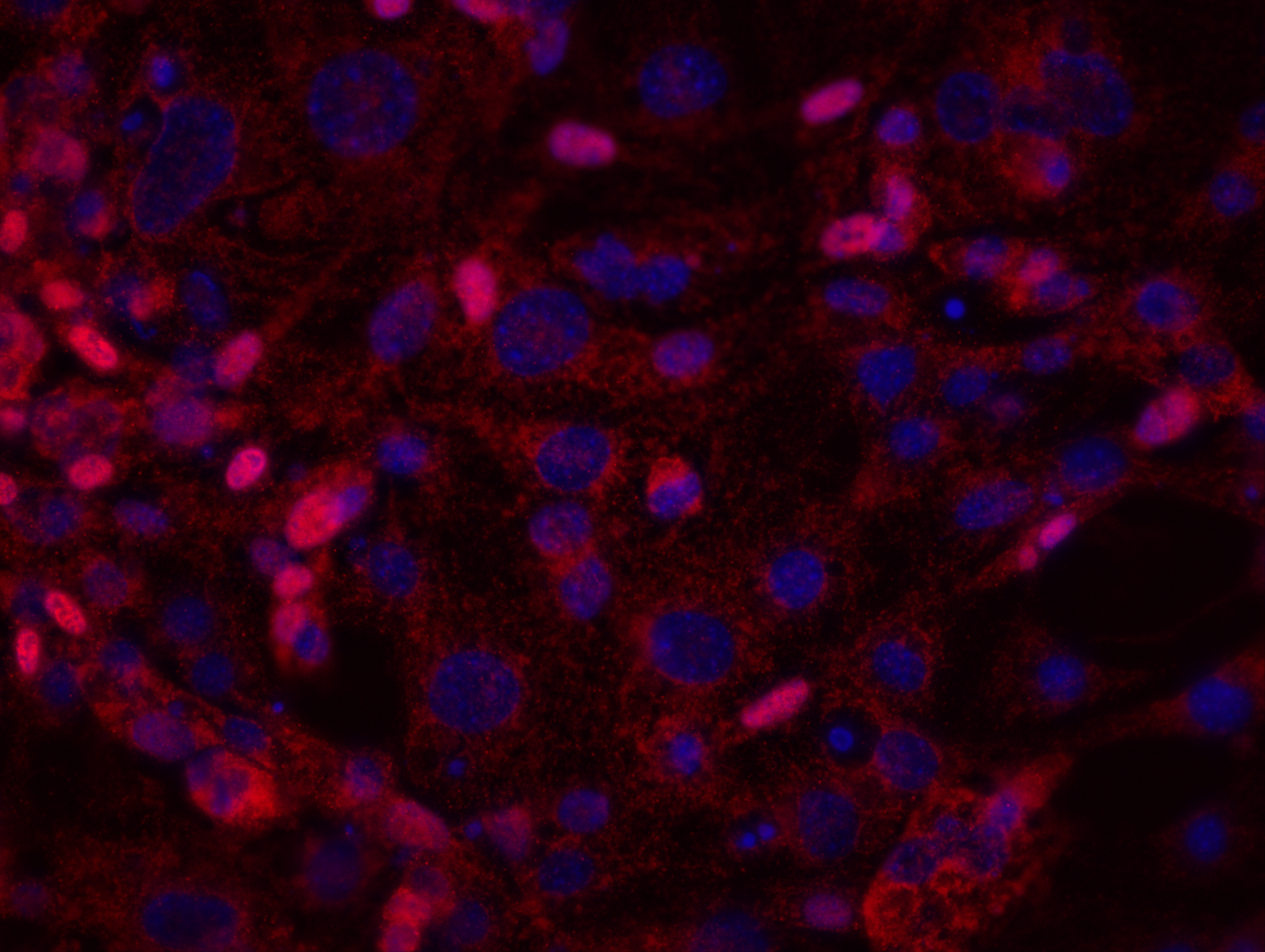

Supplement: Supplementary file 1 [file cimb-43-00144-s001.zip › cimb-1454926-supplementary/Ppar/S1/2.tif]

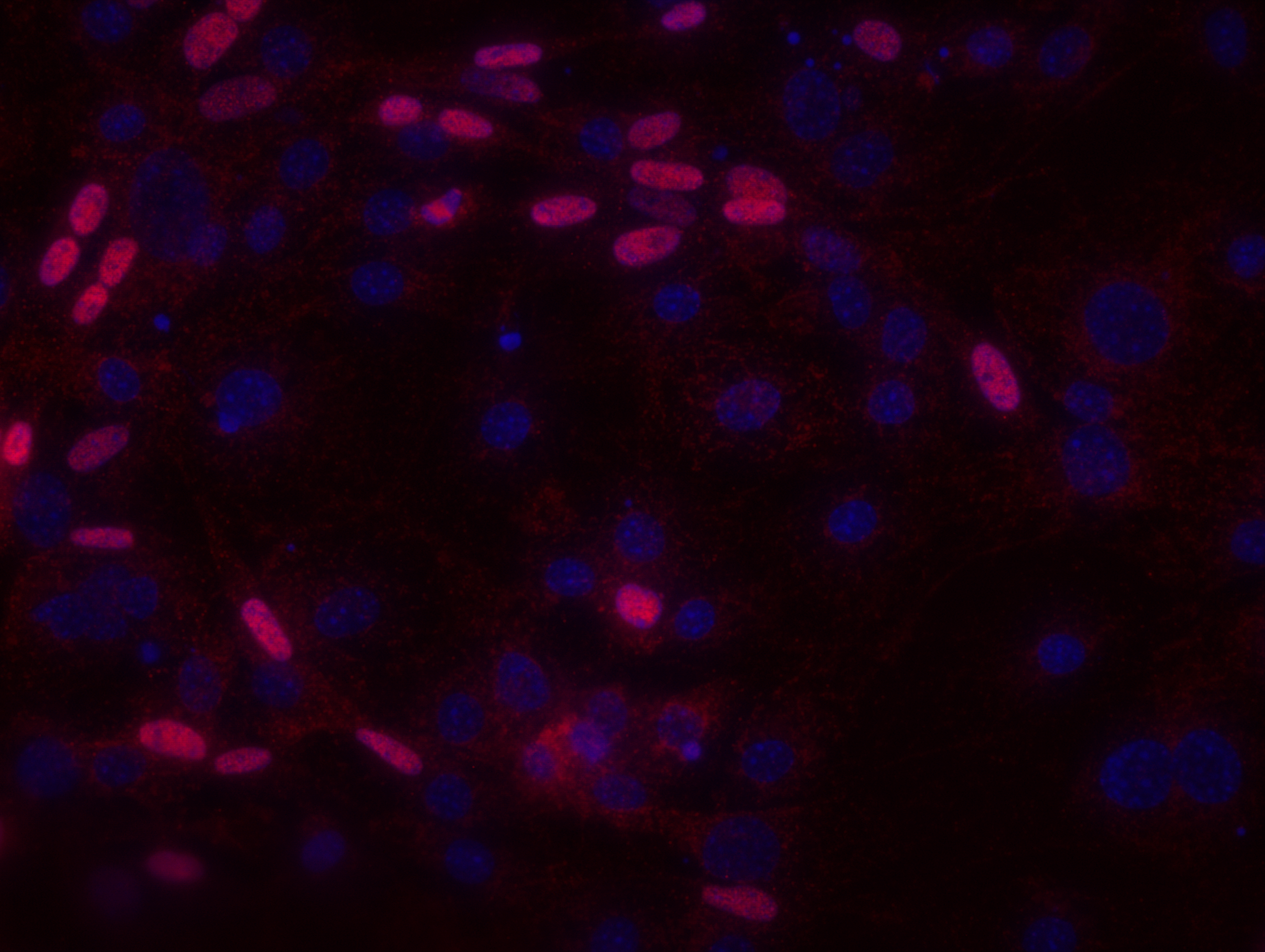

Supplement: Supplementary file 1 [file cimb-43-00144-s001.zip › cimb-1454926-supplementary/Ppar/S1/3.tif]

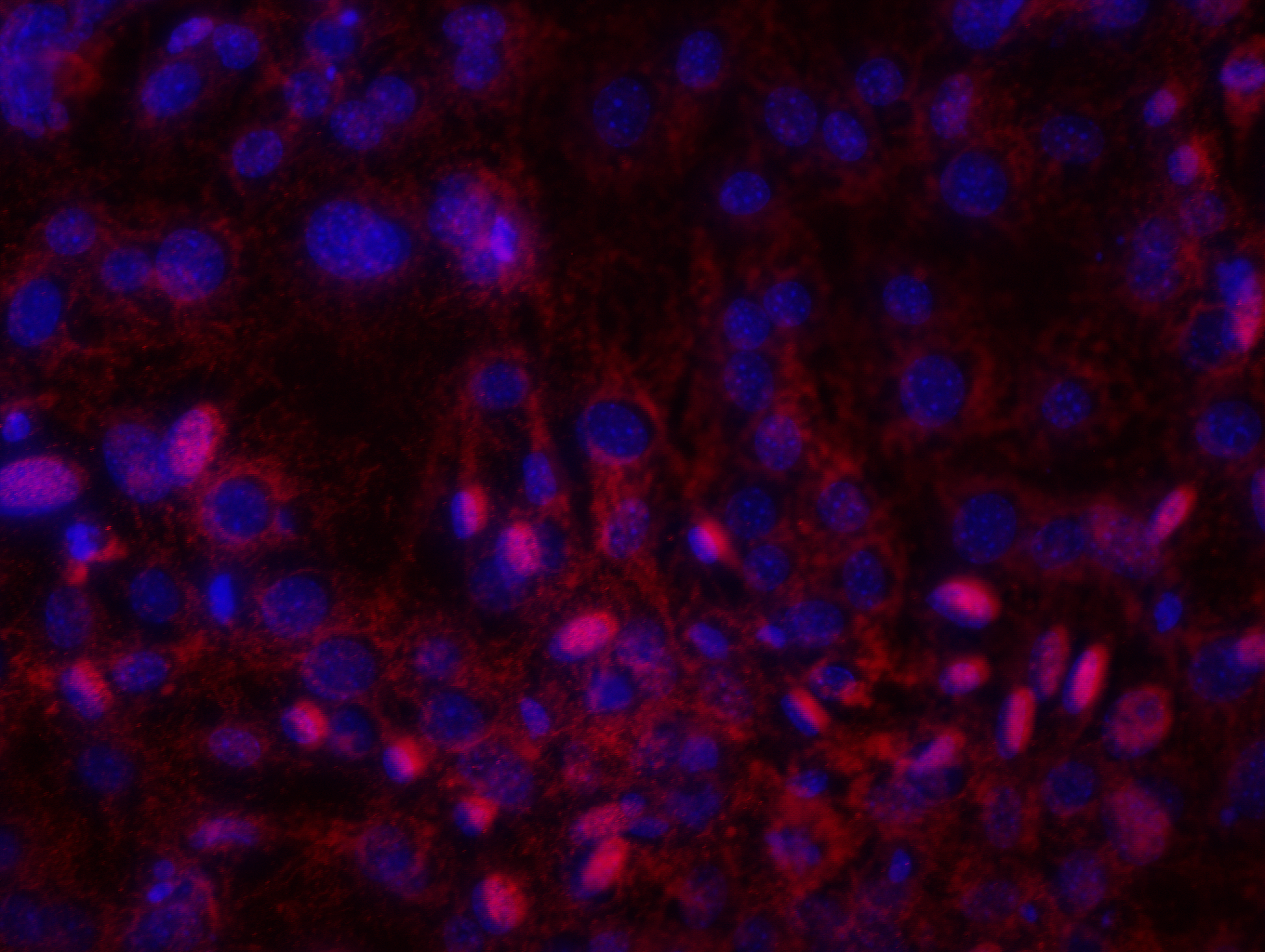

Supplement: Supplementary file 1 [file cimb-43-00144-s001.zip › cimb-1454926-supplementary/Ppar/S1/4.tif]

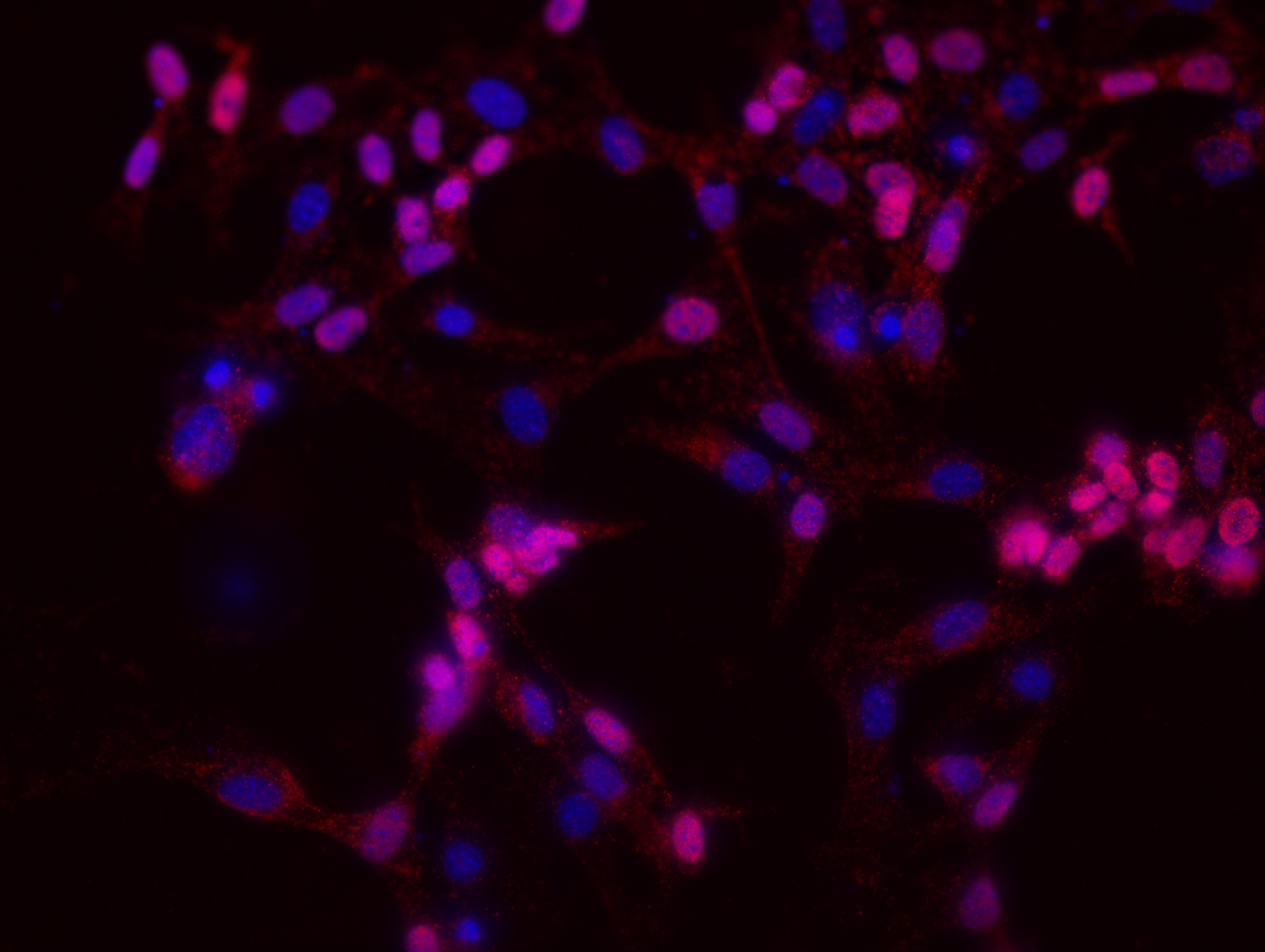

Supplement: Supplementary file 1 [file cimb-43-00144-s001.zip › cimb-1454926-supplementary/Ppar/S2/1.tif]

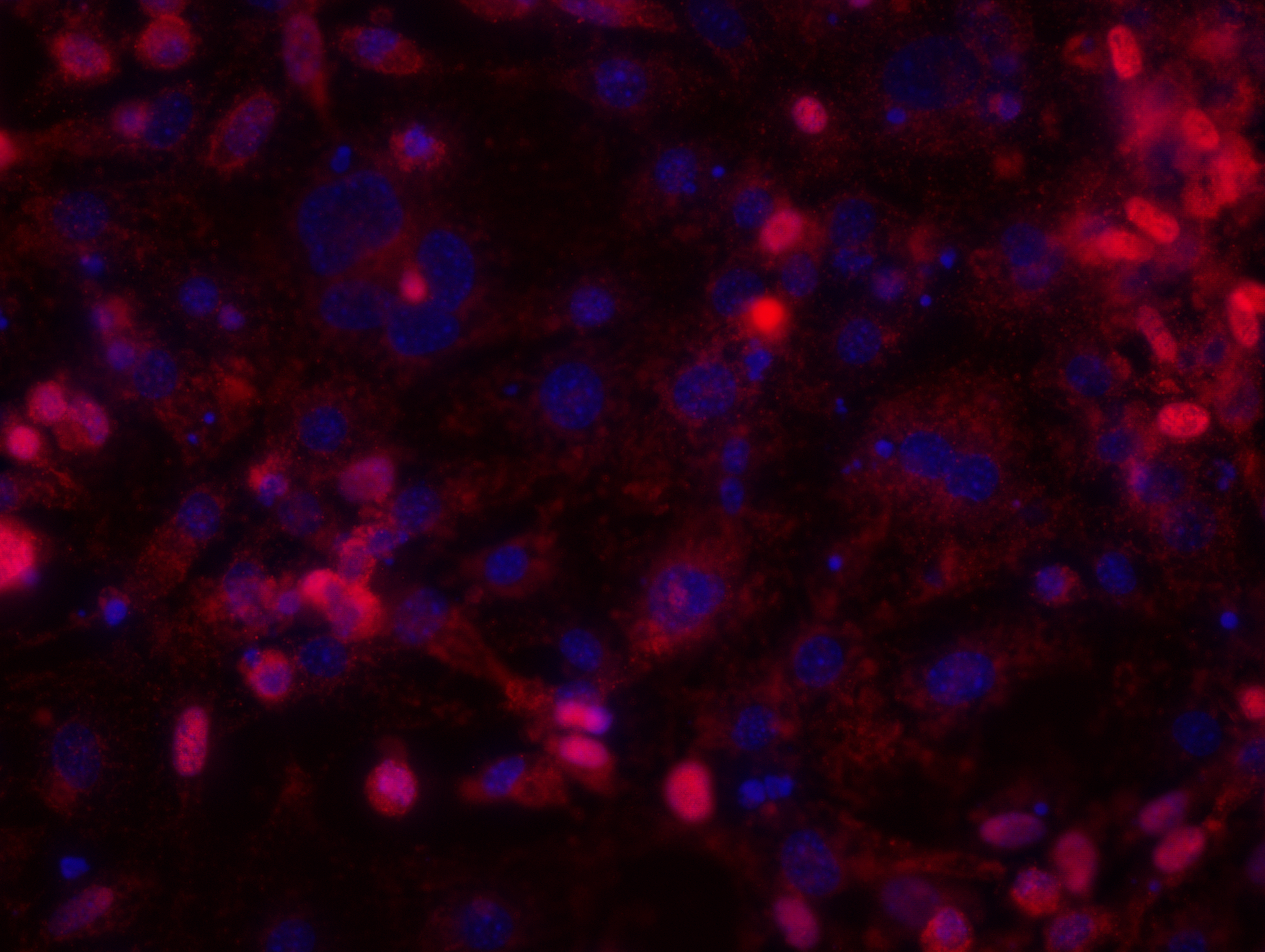

Supplement: Supplementary file 1 [file cimb-43-00144-s001.zip › cimb-1454926-supplementary/Ppar/S2/2.tif]

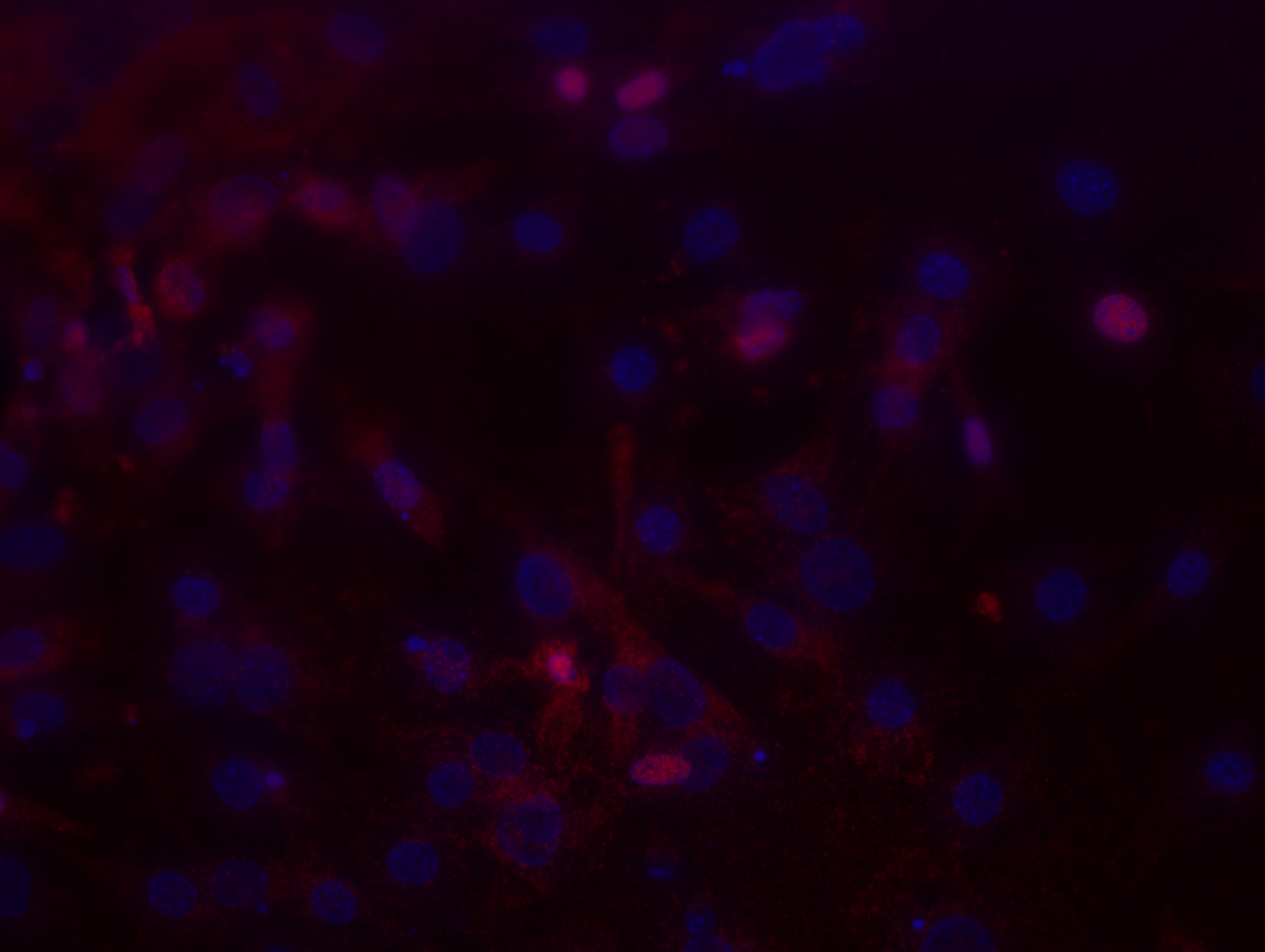

Supplement: Supplementary file 1 [file cimb-43-00144-s001.zip › cimb-1454926-supplementary/Ppar/S2/3.tif]

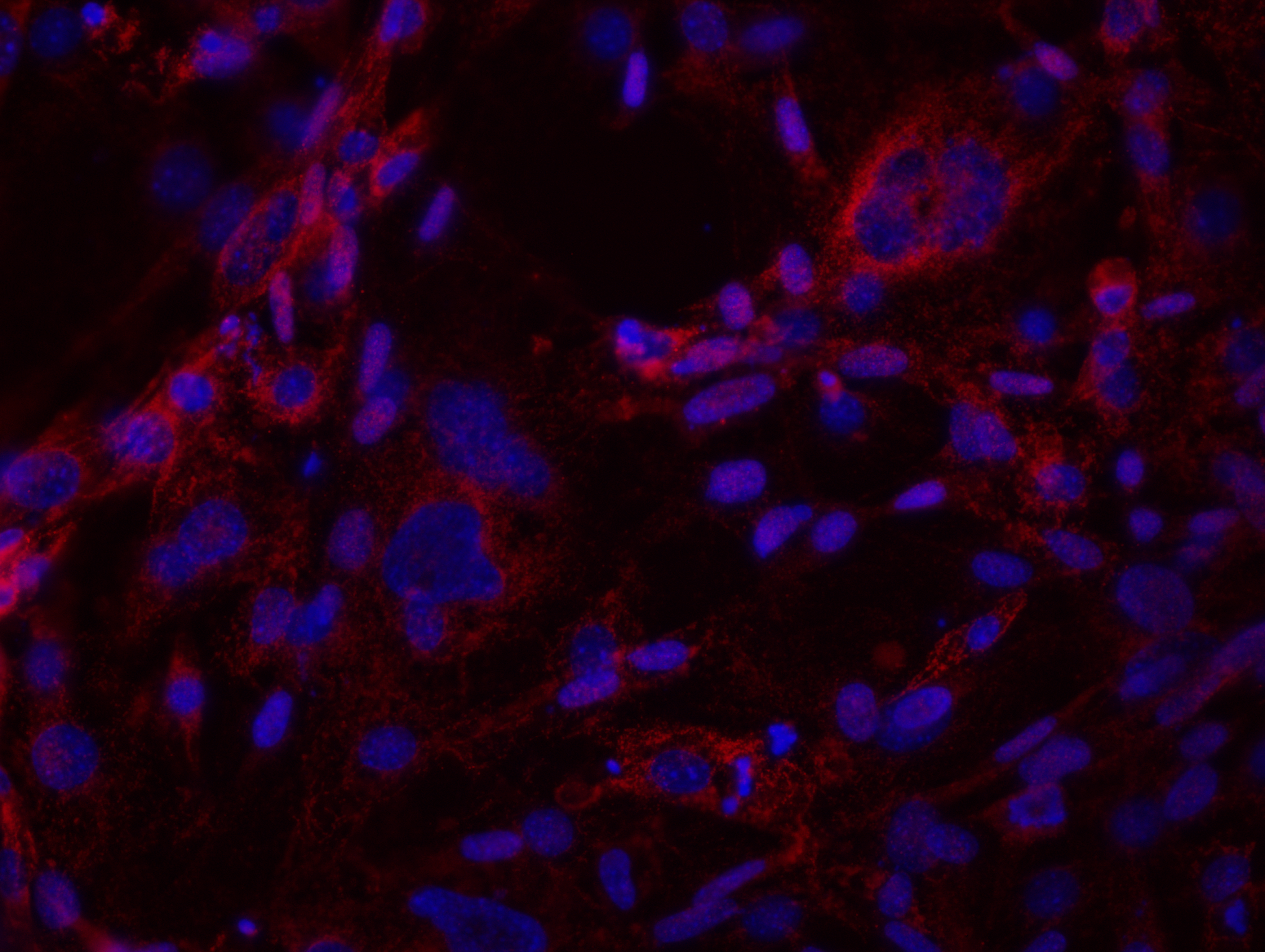

Supplement: Supplementary file 1 [file cimb-43-00144-s001.zip › cimb-1454926-supplementary/Ppar/S2/4.tif]

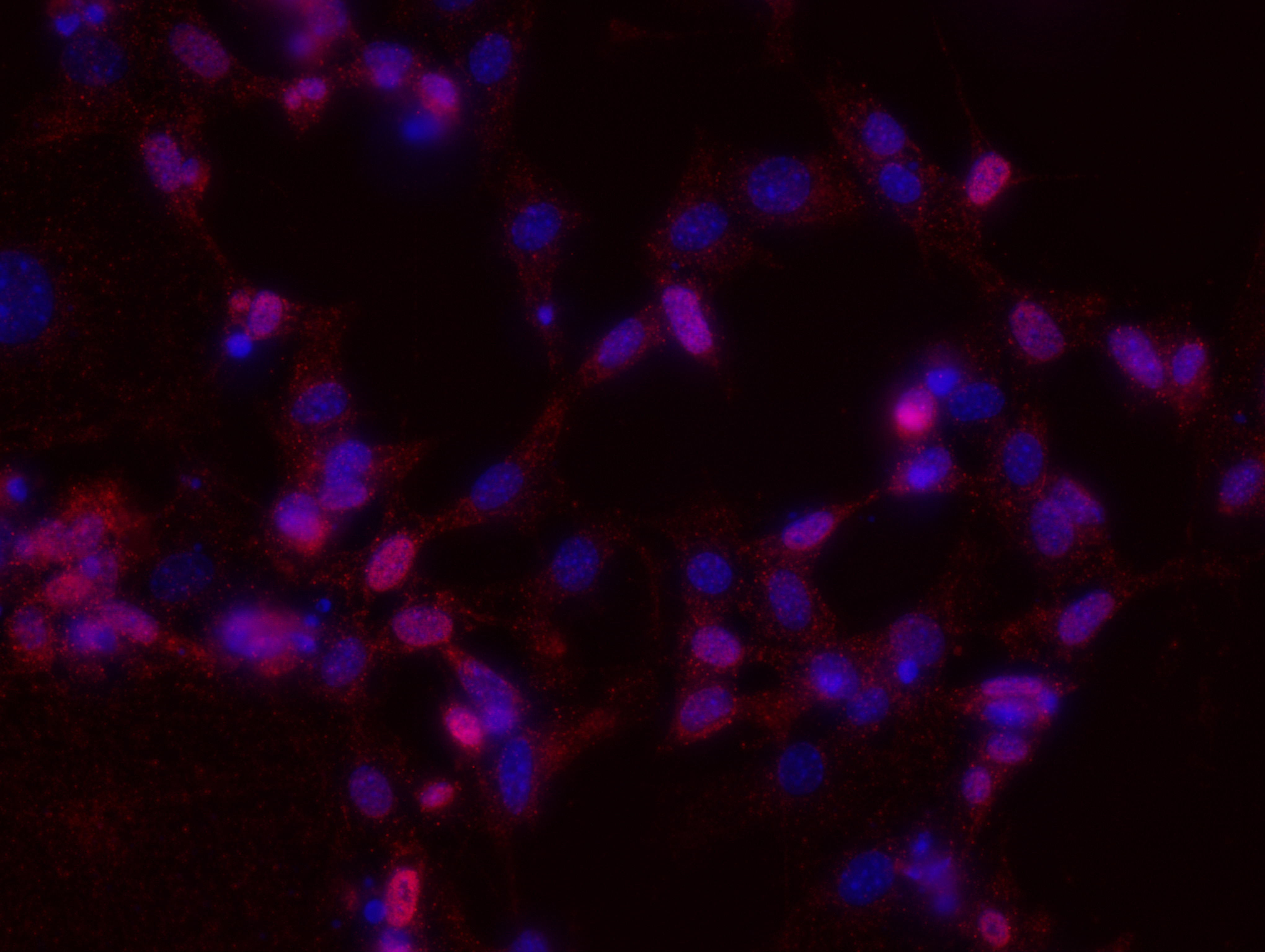

Supplement: Supplementary file 1 [file cimb-43-00144-s001.zip › cimb-1454926-supplementary/Ppar/S3/1.tif]

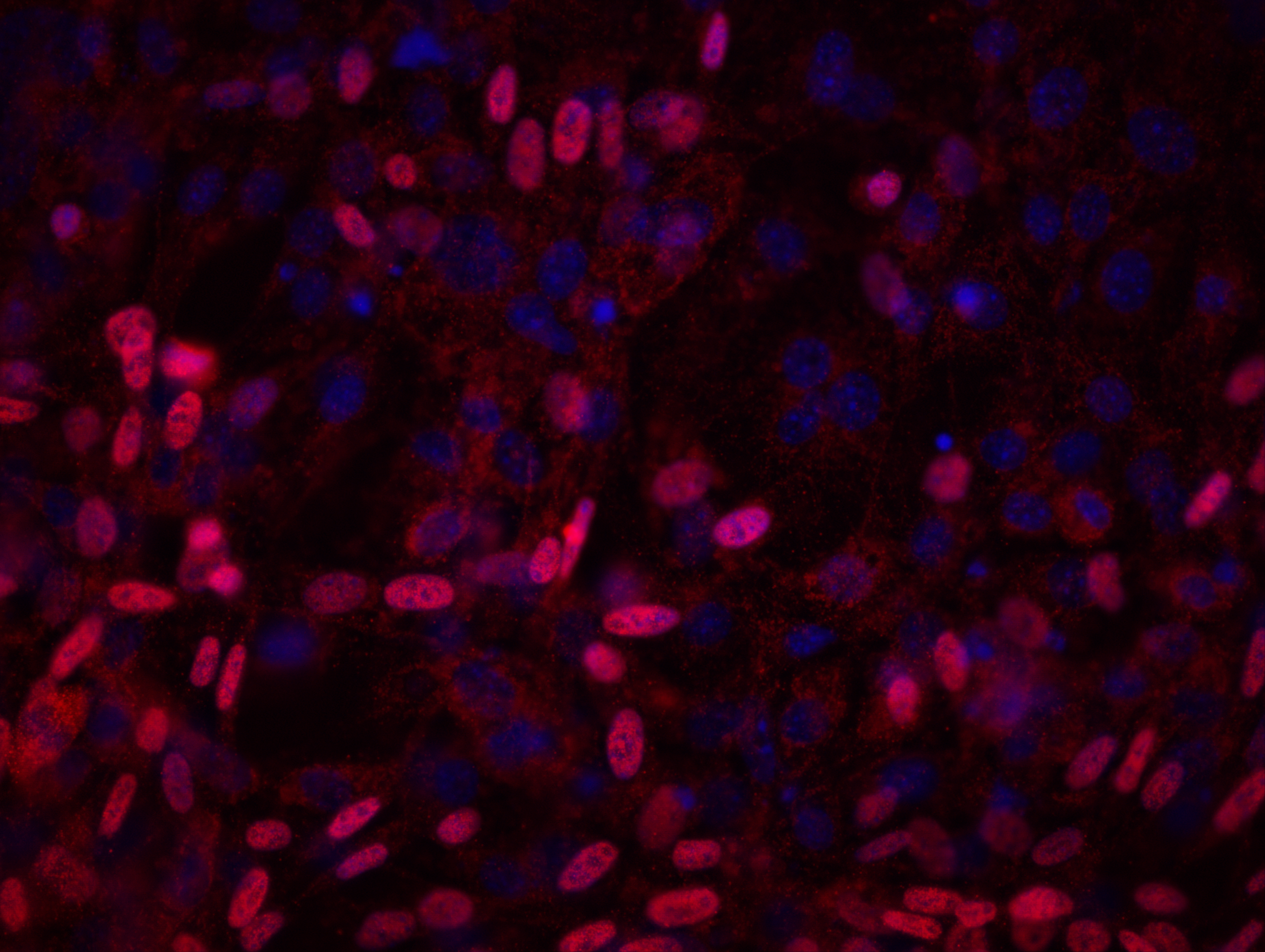

Supplement: Supplementary file 1 [file cimb-43-00144-s001.zip › cimb-1454926-supplementary/Ppar/S3/2.tif]

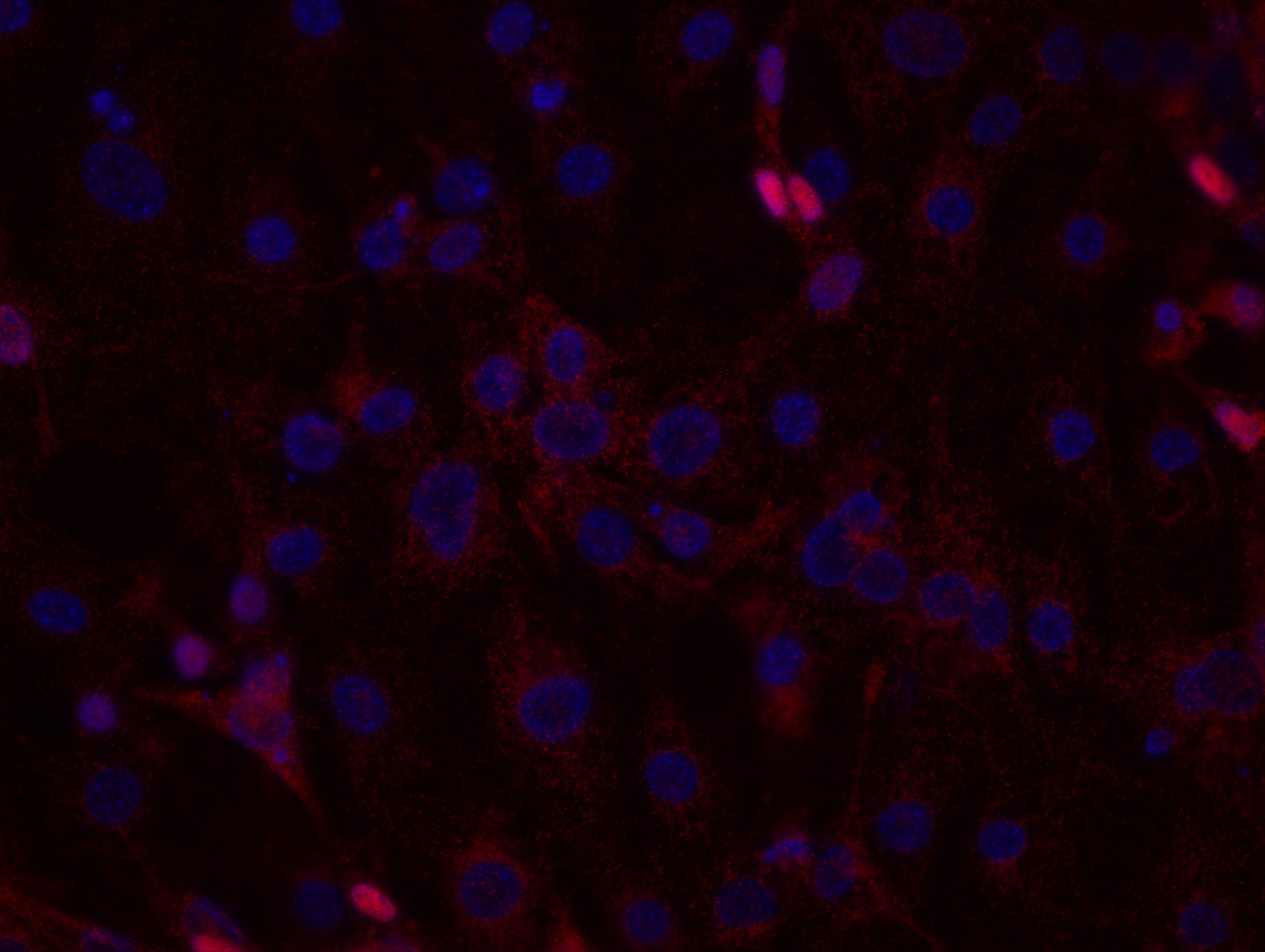

Supplement: Supplementary file 1 [file cimb-43-00144-s001.zip › cimb-1454926-supplementary/Ppar/S3/3.tif]

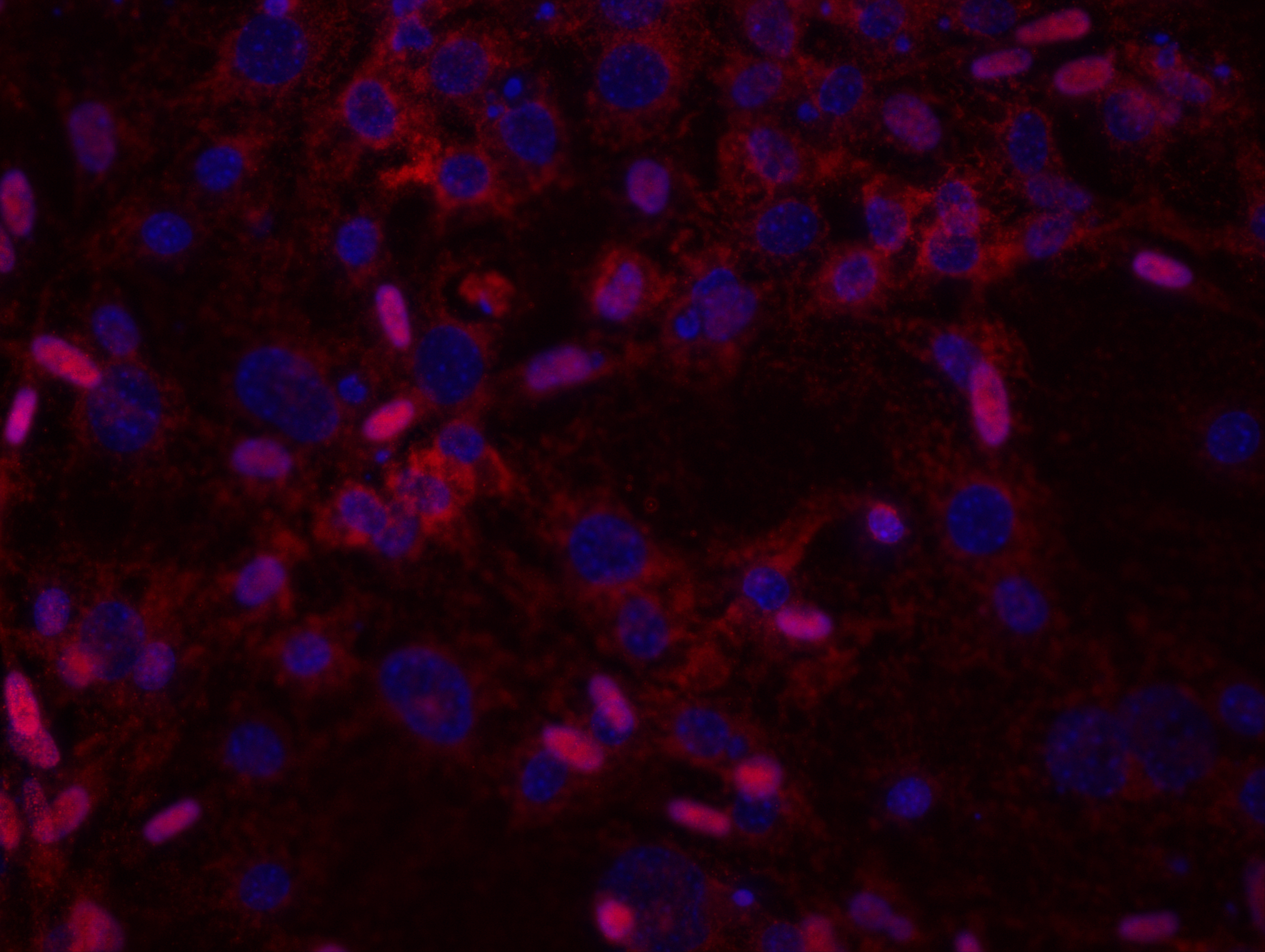

Supplement: Supplementary file 1 [file cimb-43-00144-s001.zip › cimb-1454926-supplementary/Ppar/S3/4.tif]

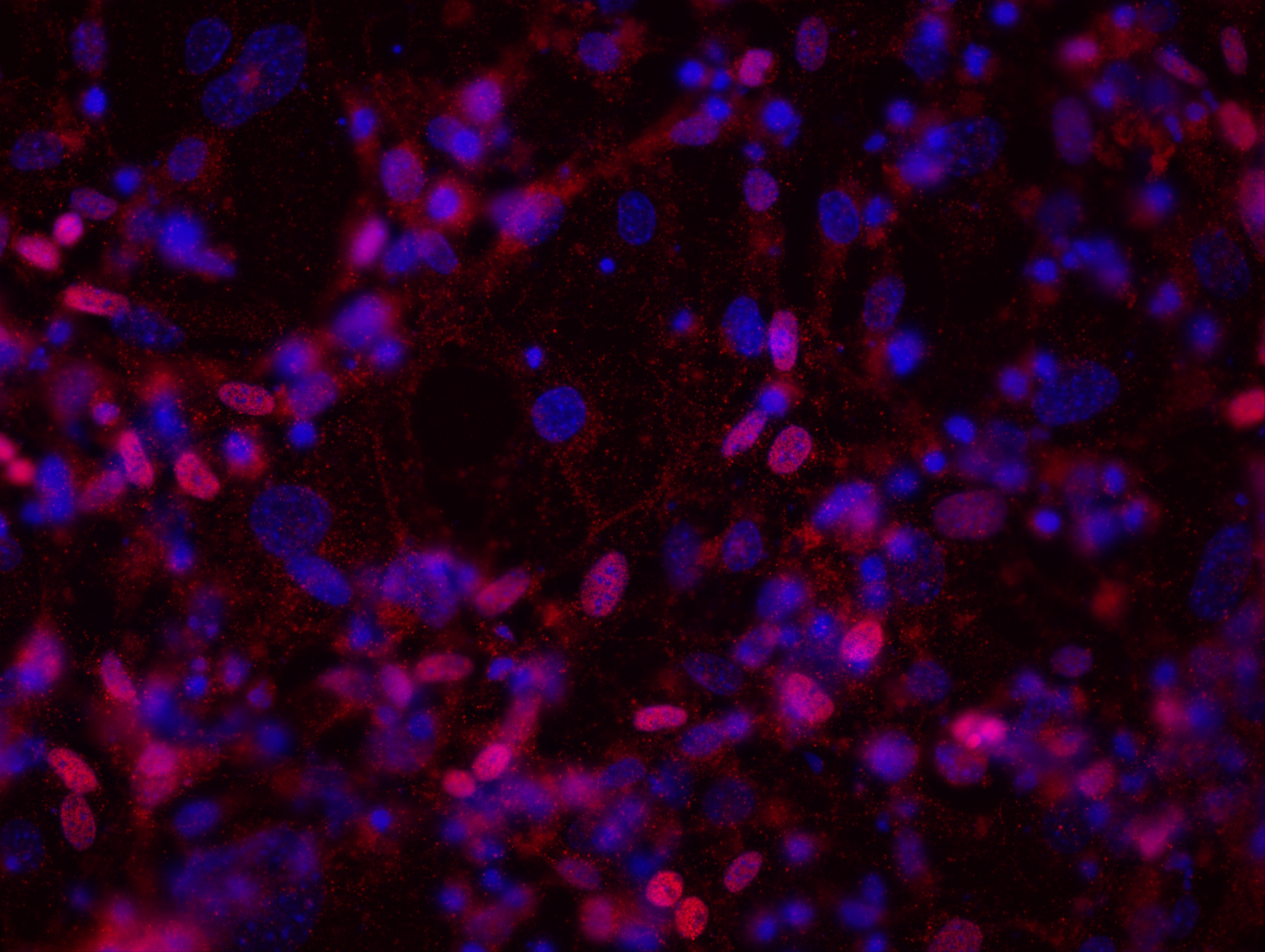

Supplement: Supplementary file 1 [file cimb-43-00144-s001.zip › cimb-1454926-supplementary/Ppar/SM1/1.tif]

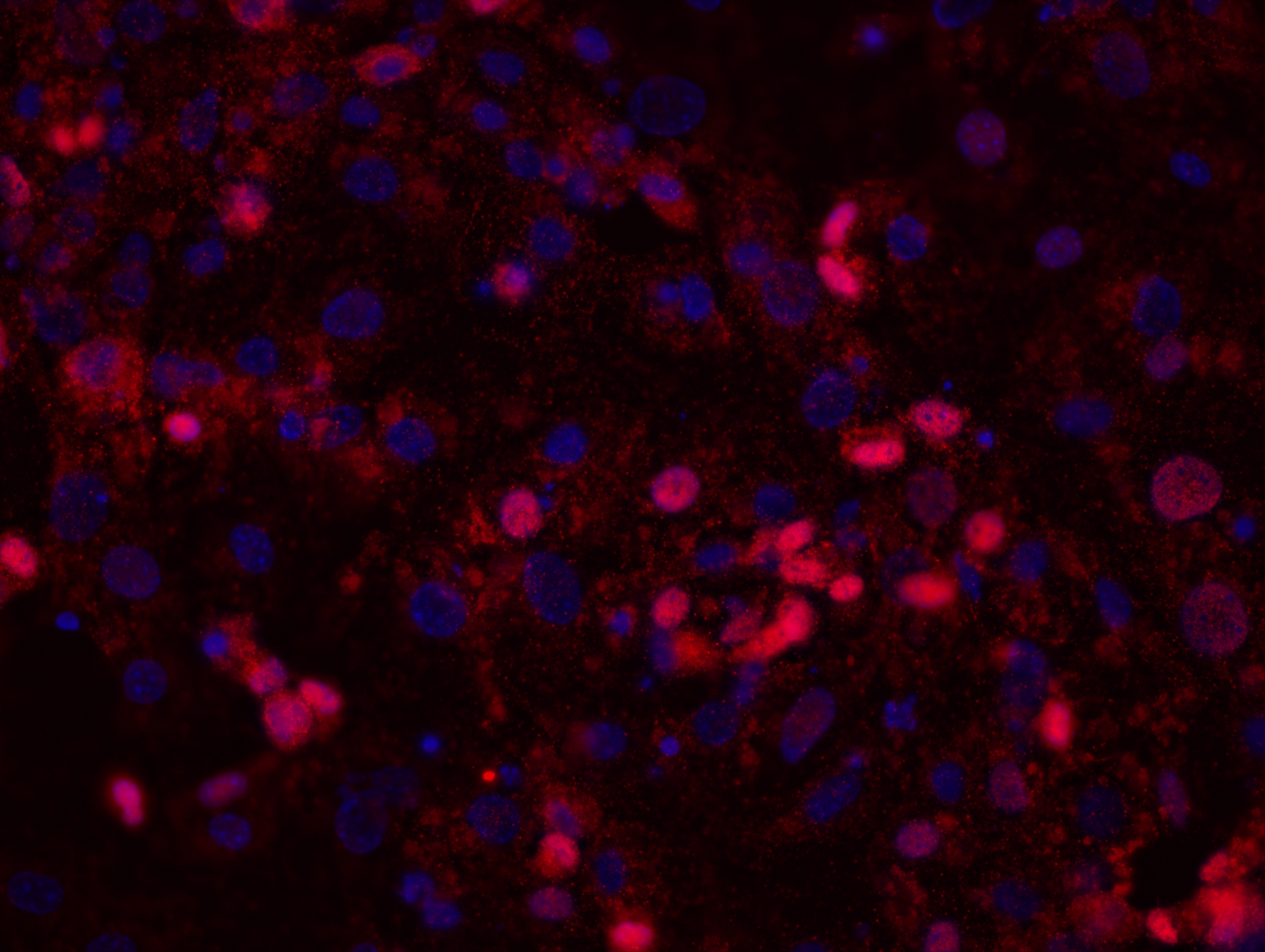

Supplement: Supplementary file 1 [file cimb-43-00144-s001.zip › cimb-1454926-supplementary/Ppar/SM1/2.tif]

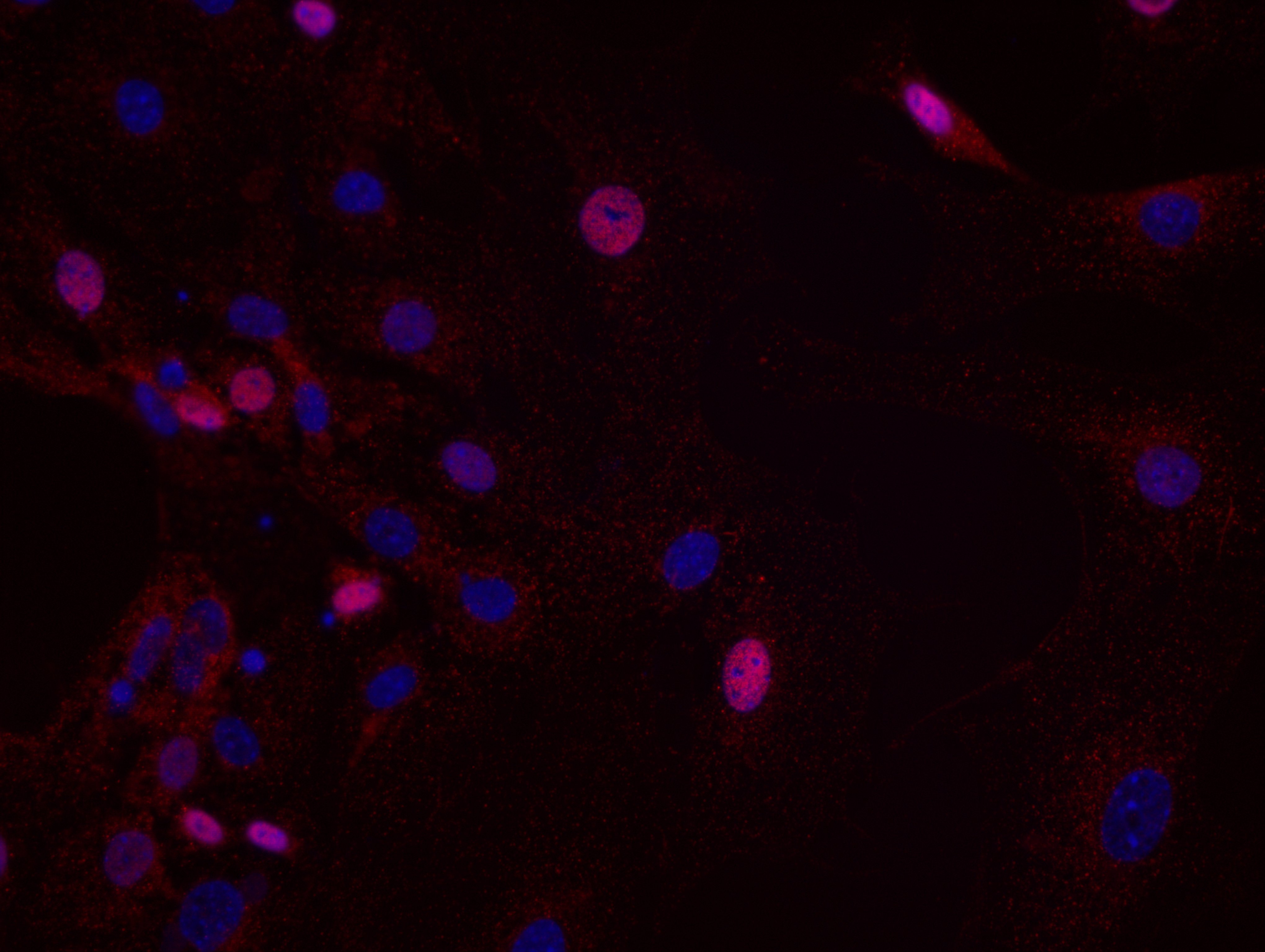

Supplement: Supplementary file 1 [file cimb-43-00144-s001.zip › cimb-1454926-supplementary/Ppar/SM1/3.tif]

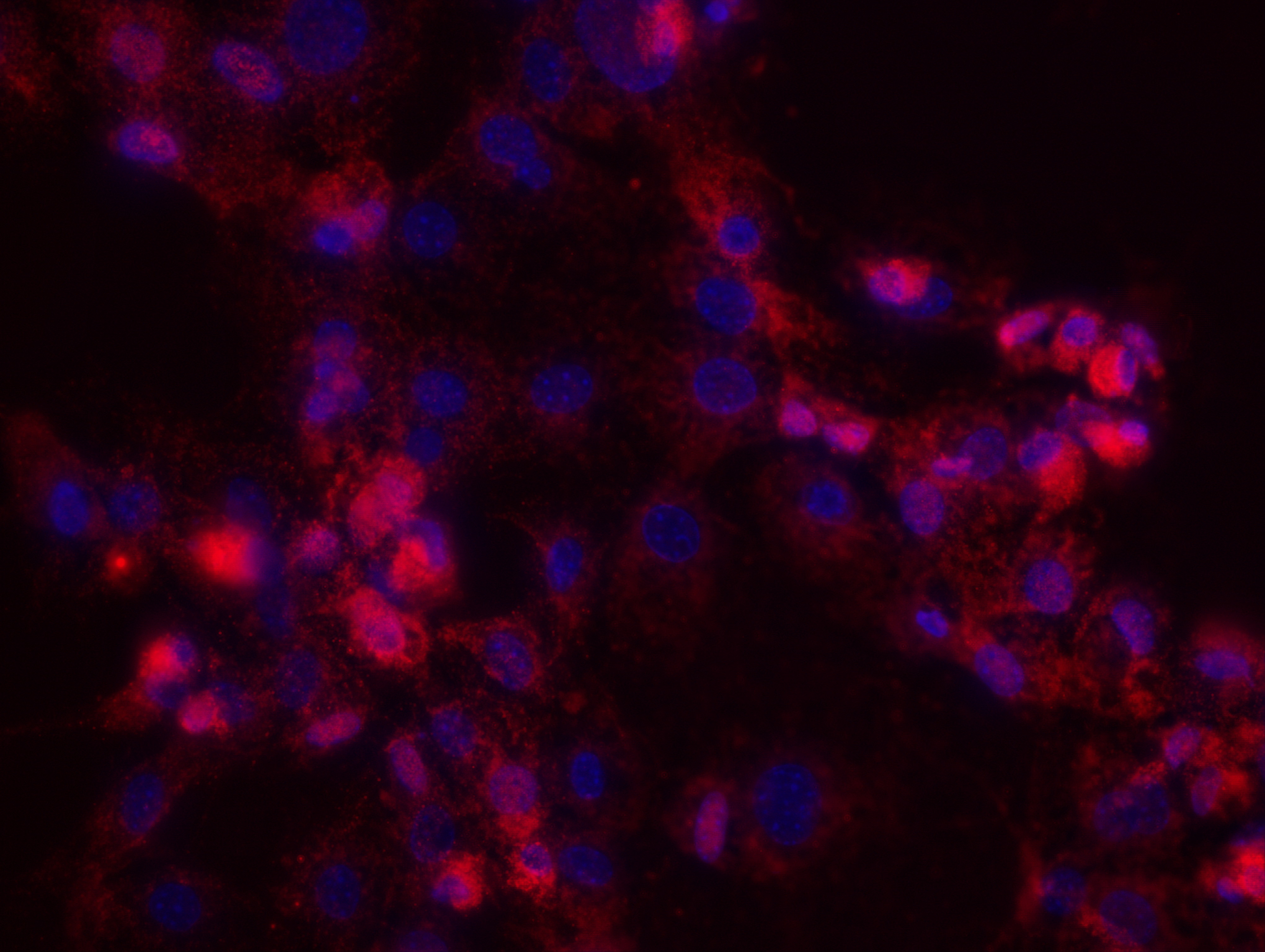

Supplement: Supplementary file 1 [file cimb-43-00144-s001.zip › cimb-1454926-supplementary/Ppar/SM1/4.tif]

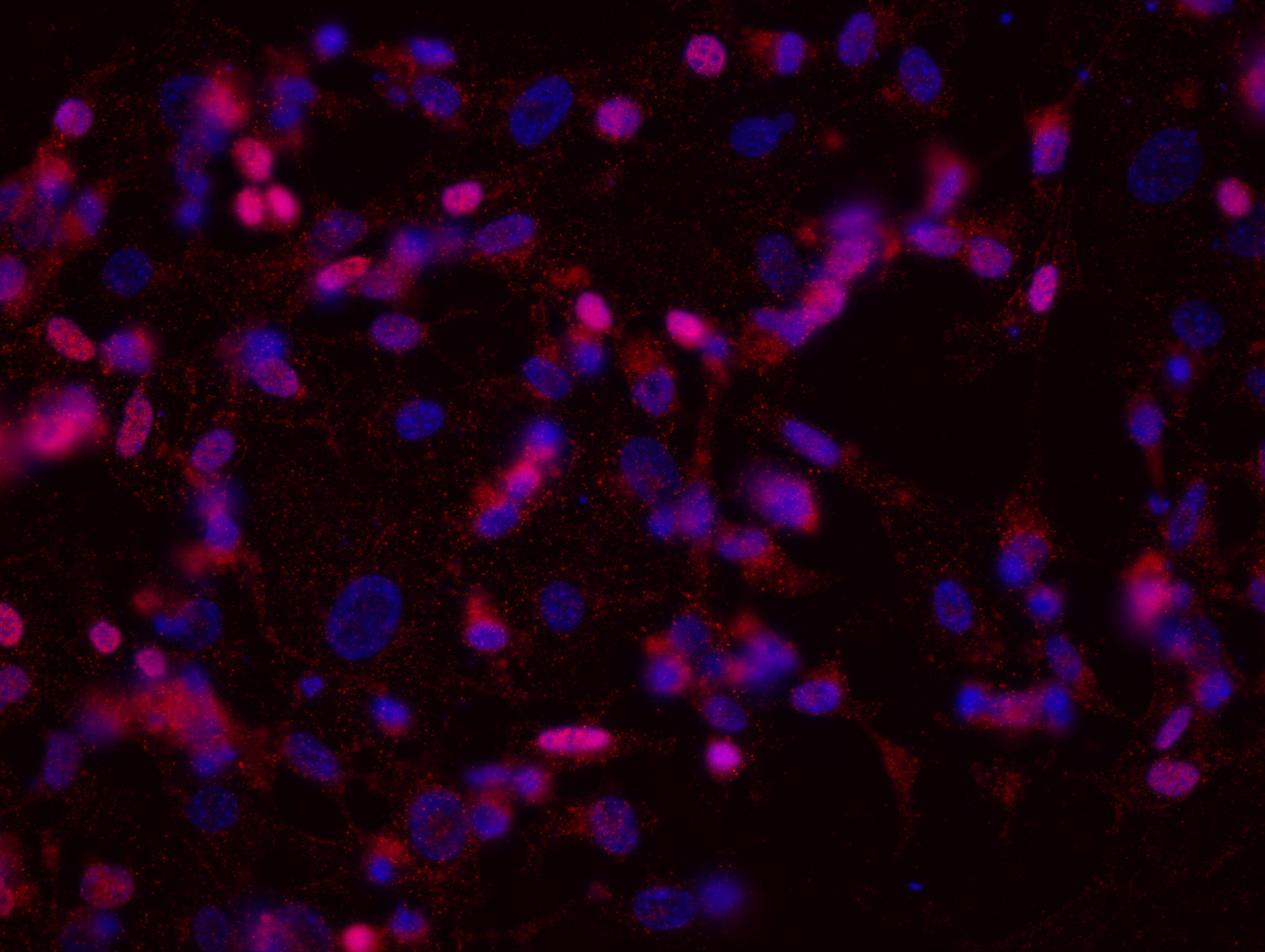

Supplement: Supplementary file 1 [file cimb-43-00144-s001.zip › cimb-1454926-supplementary/Ppar/SM2/10_PPAR_grA_SM2.tif]

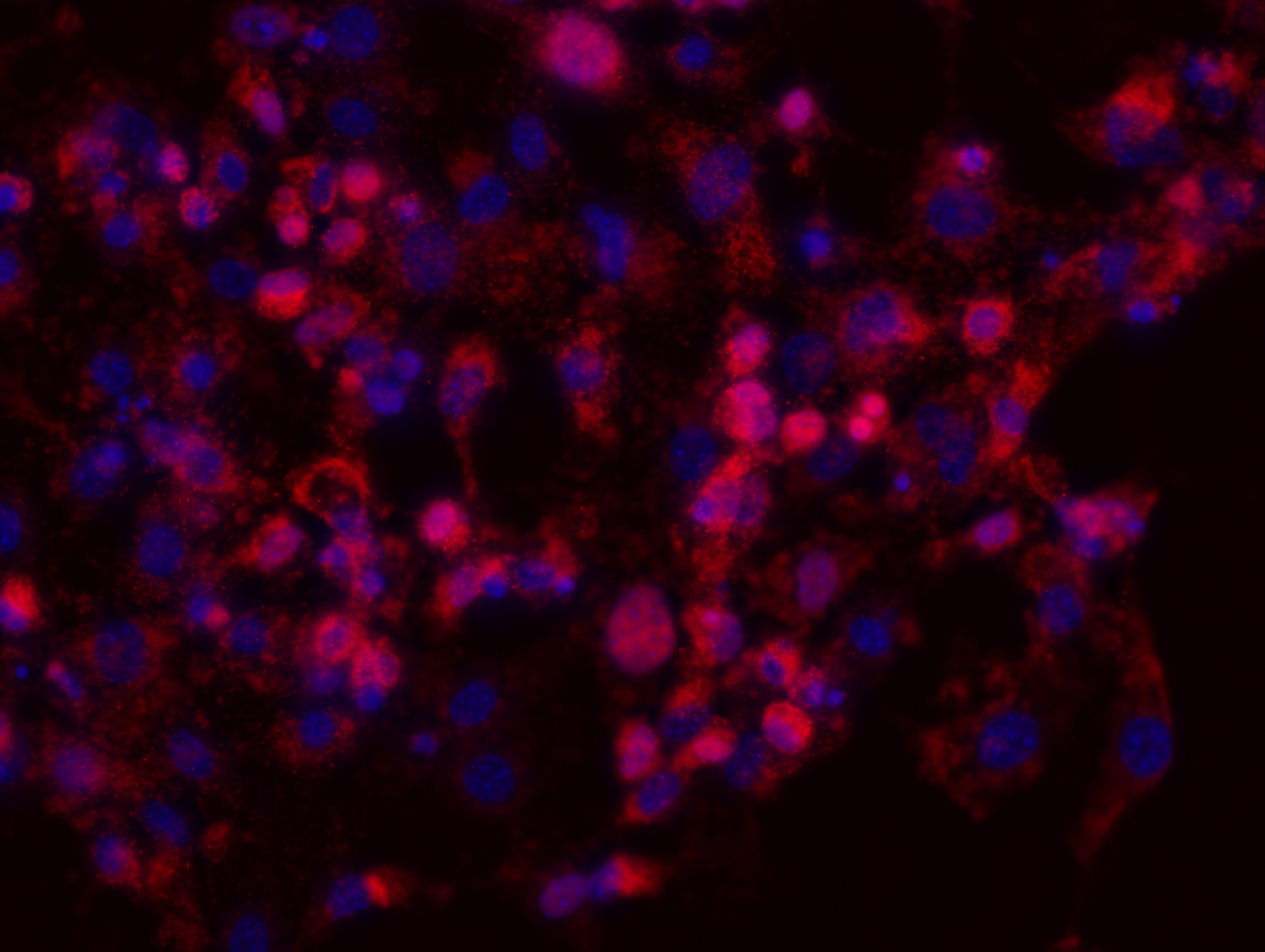

Supplement: Supplementary file 1 [file cimb-43-00144-s001.zip › cimb-1454926-supplementary/Ppar/SM2/21_PPAR_grB_SM2.tif]

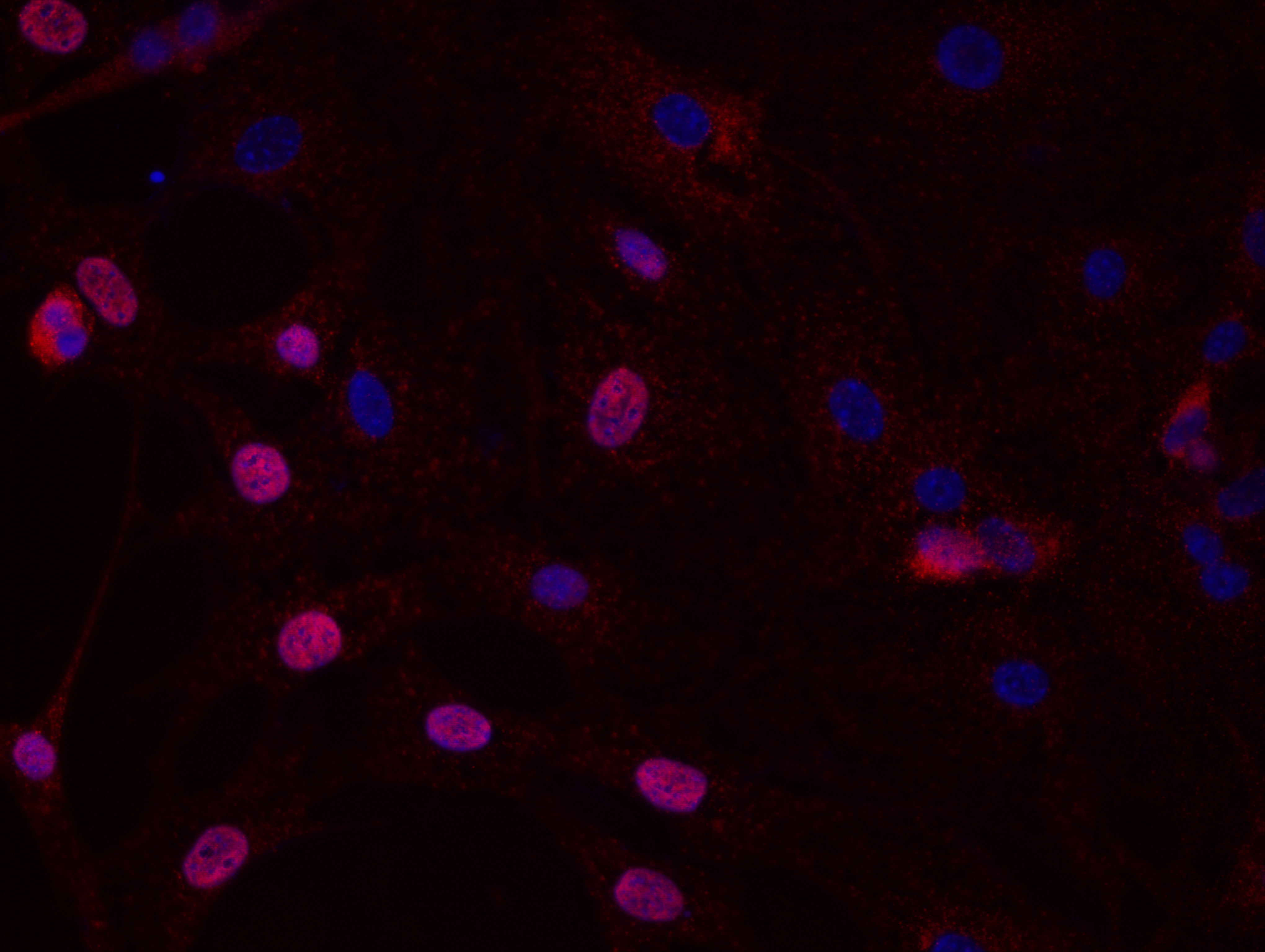

Supplement: Supplementary file 1 [file cimb-43-00144-s001.zip › cimb-1454926-supplementary/Ppar/SM2/32_PPAR_grC_SM2.tif]

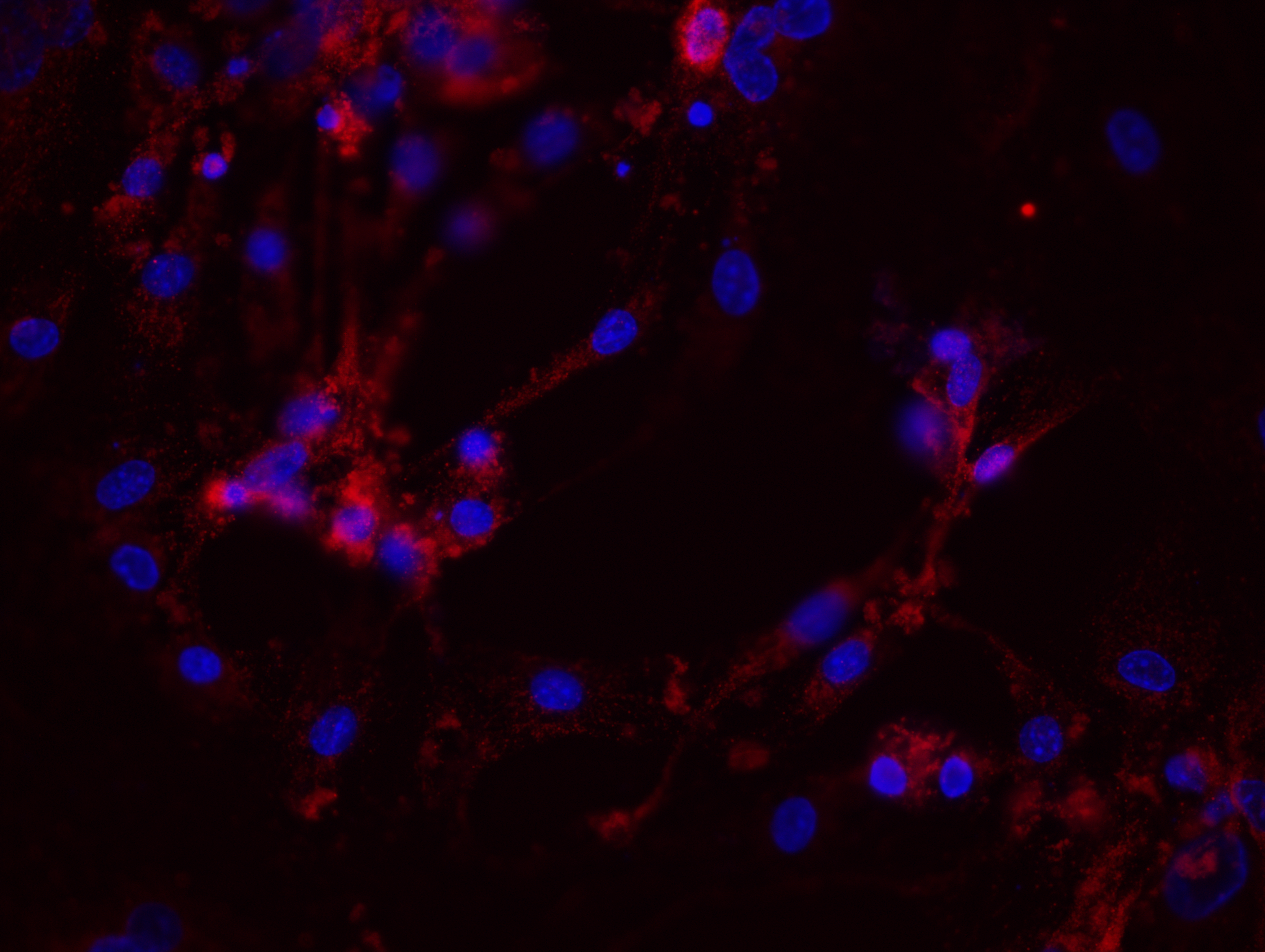

Supplement: Supplementary file 1 [file cimb-43-00144-s001.zip › cimb-1454926-supplementary/Ppar/SM2/43_PPAR_grD_SM2.tif]

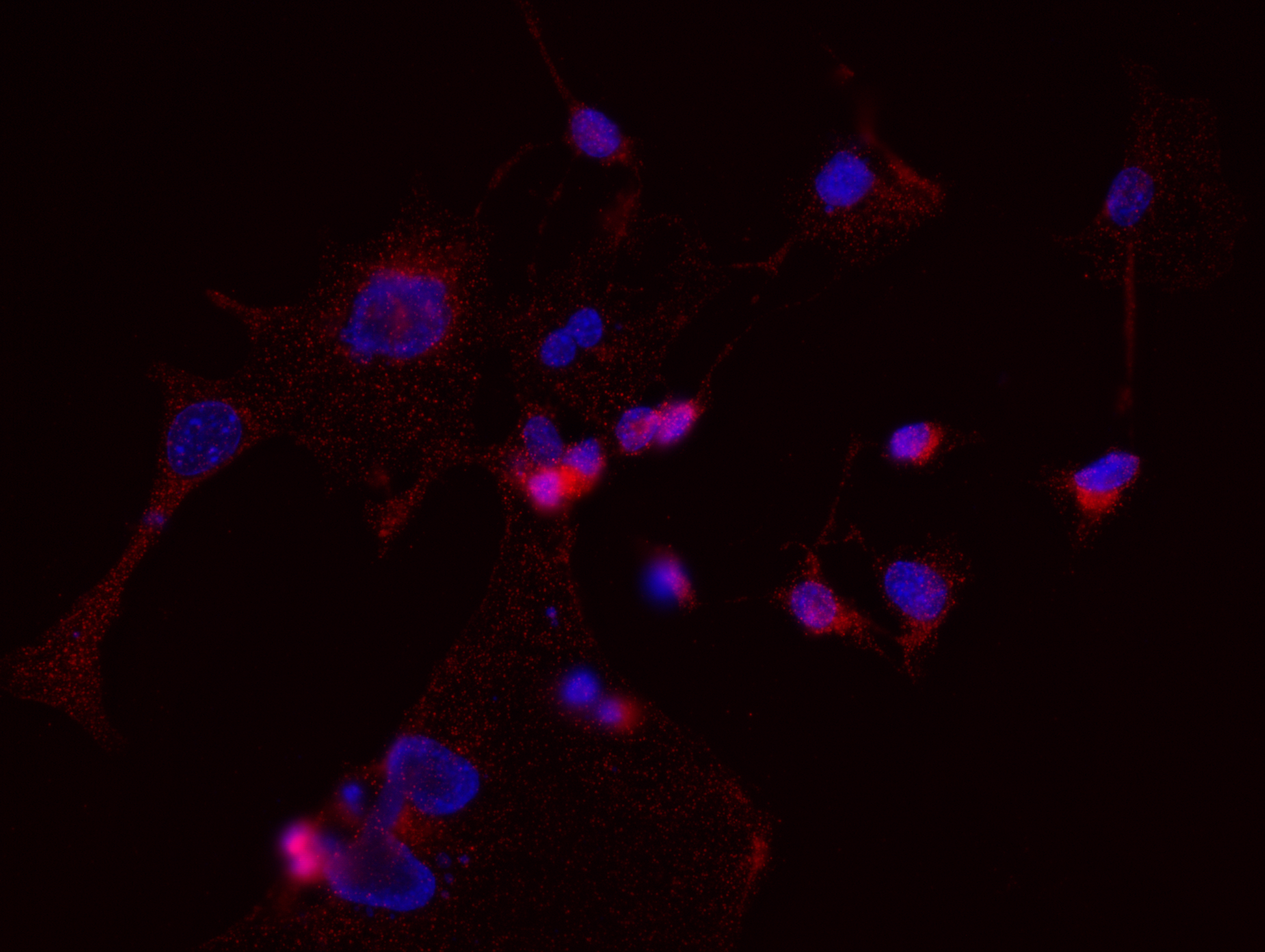

Supplement: Supplementary file 1 [file cimb-43-00144-s001.zip › cimb-1454926-supplementary/Ppar/SM3/11_PPAR_grA_SM3.tif]

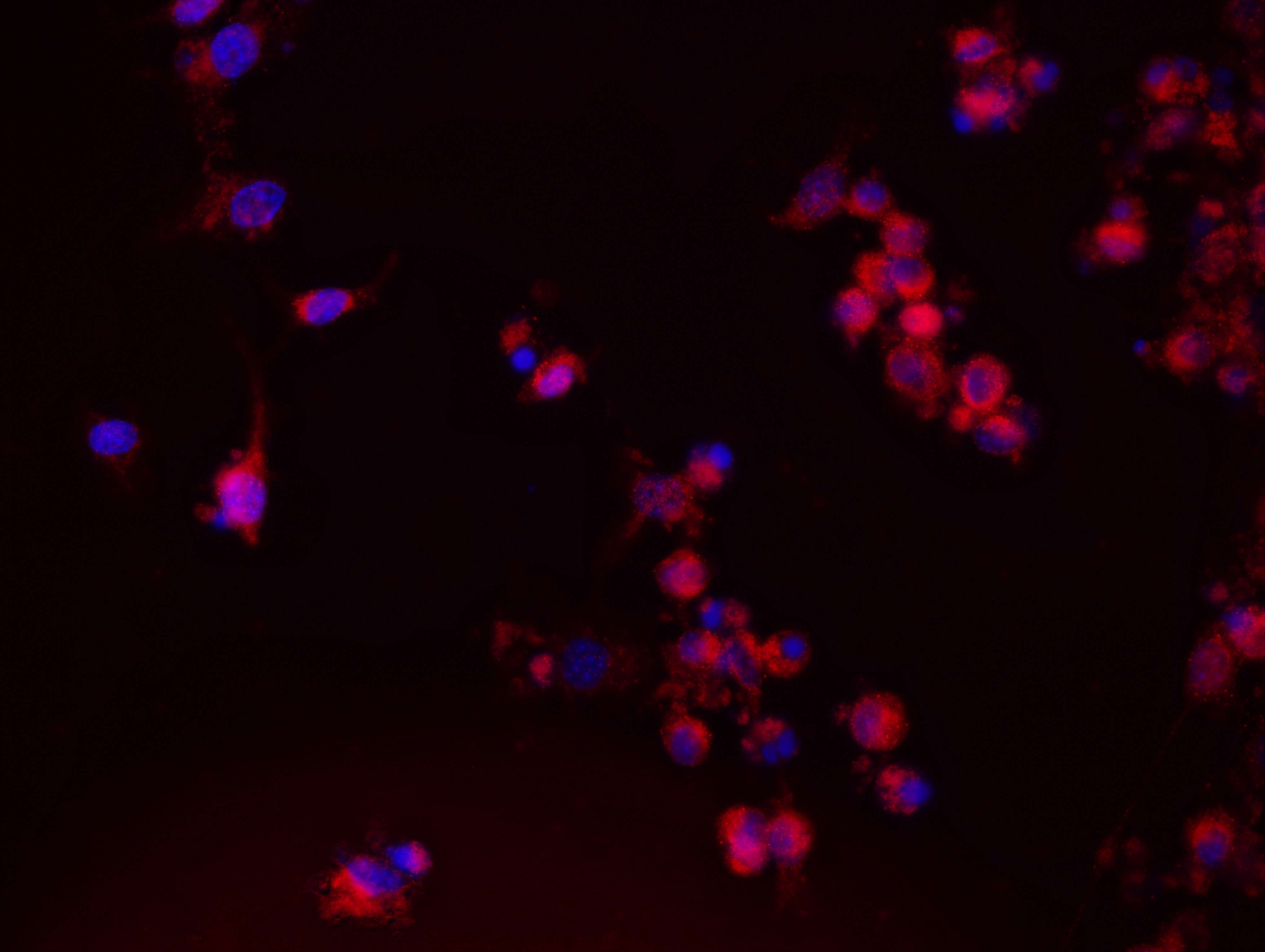

Supplement: Supplementary file 1 [file cimb-43-00144-s001.zip › cimb-1454926-supplementary/Ppar/SM3/22_PPAR_grB_SM3.tif]

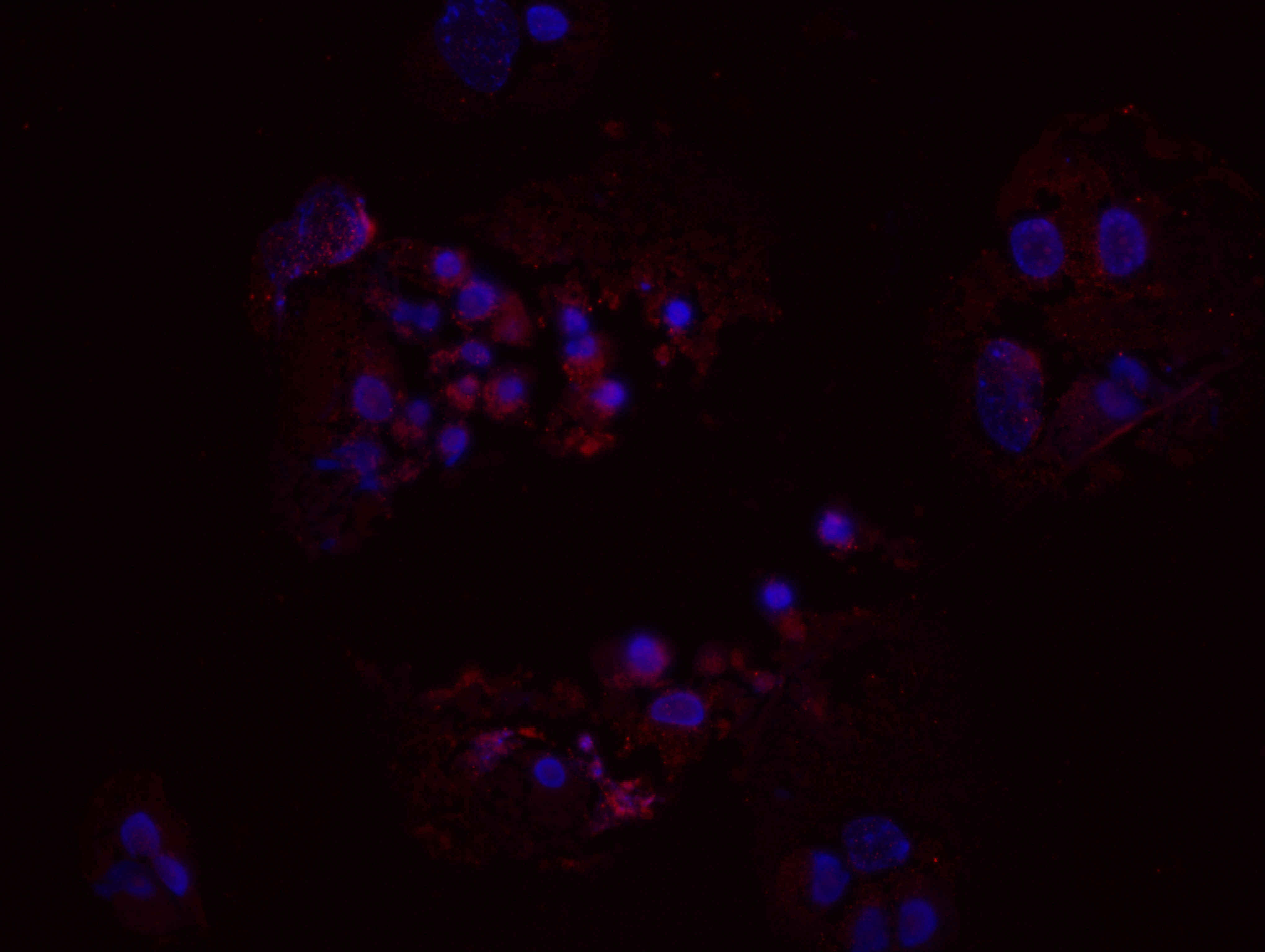

Supplement: Supplementary file 1 [file cimb-43-00144-s001.zip › cimb-1454926-supplementary/Ppar/SM3/33_PPAR_grC_SM3.tif]

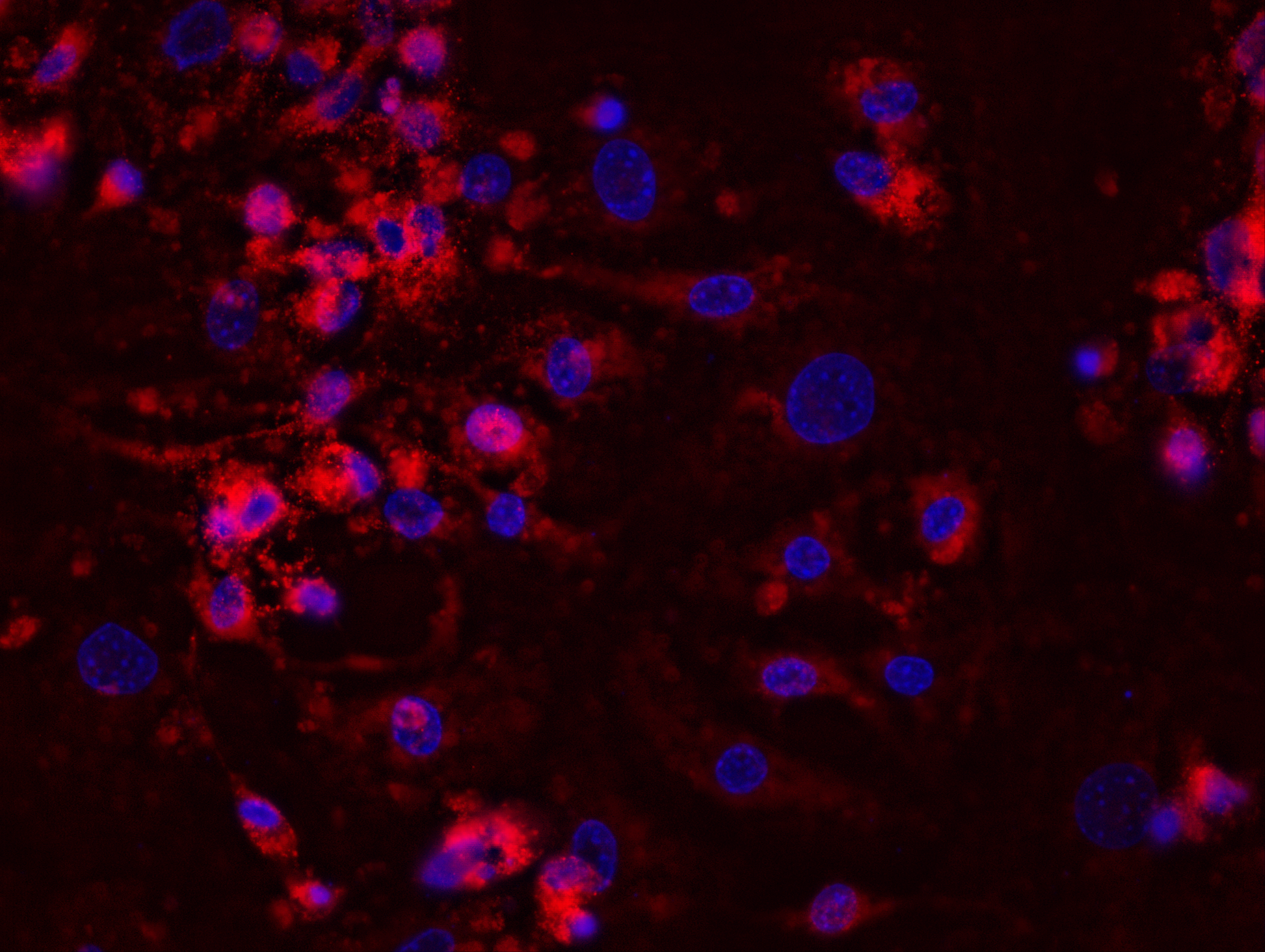

Supplement: Supplementary file 1 [file cimb-43-00144-s001.zip › cimb-1454926-supplementary/Ppar/SM3/44_PPAR_grD_SM3.tif]
